# Modelling a Human Blood-Brain Barrier Co-Culture Using an Ultrathin Silicon Nitride Membrane-Based Microfluidic Device

**DOI:** 10.3390/ijms24065624

**Published:** 2023-03-15

**Authors:** Diana Hudecz, Molly C. McCloskey, Sandra Vergo, Søren Christensen, James L. McGrath, Morten S. Nielsen

**Affiliations:** 1 Department of Biomedicine, Aarhus University, 8000 Aarhus, Denmark; 2 Department of Biomedical Engineering, University of Rochester, Rochester, NY 14627, USA; 3 Biotherapeutic Discovery, H. Lundbeck A/S, Valby, 2500 Copenhagen, Denmark

**Keywords:** blood-brain barrier, stem cells, monoclonal antibodies, tissue chips, microscopy

## Abstract

Understanding the vesicular trafficking of receptors and receptor ligands in the brain capillary endothelium is essential for the development of the next generations of biologics targeting neurodegenerative diseases. Such complex biological questions are often approached by in vitro models in combination with various techniques. Here, we present the development of a stem cell-based human in vitro blood-brain barrier model composed of induced brain microvascular endothelial cells (iBMECs) on the modular µSiM (a microdevice featuring a silicon nitride membrane) platform. The µSiM was equipped with a 100 nm thick nanoporous silicon nitride membrane with glass-like imaging quality that allowed the use of high-resolution in situ imaging to study the intracellular trafficking. As a proof-of-concept experiment, we investigated the trafficking of two monoclonal antibodies (mAb): an anti-human transferrin receptor mAb (15G11) and an anti-basigin mAb (#52) using the µSiM-iBMEC-human astrocyte model. Our results demonstrated effective endothelial uptake of the selected antibodies; however, no significant transcytosis was observed when the barrier was tight. In contrast, when the iBMECs did not form a confluent barrier on the µSiM, the antibodies accumulated inside both the iBMECs and astrocytes, demonstrating that the cells have an active endocytic and subcellular sorting machinery and that the µSiM itself does not hinder antibody transport. In conclusion, our µSiM-iBMEC-human astrocyte model provides a tight barrier with endothelial-like cells, which can be used for high-resolution in situ imaging and for studying receptor-mediated transport and transcytosis in a physiological barrier.

## 1. Introduction

Delivery of pharmaceutical drug candidates to the brain is hindered by the blood-brain barrier (BBB), which makes the treatment of neurological and neurodegenerative diseases particularly challenging [1]. Advances in biotechnology have shifted the focus from small synthetic molecules and transcellular targeting towards large macromolecules, such as monoclonal antibodies (mAb), antibody fragments, peptides, or nanoparticles decorated with such macromolecules [2,3]. Most of these macromolecules target specific endocytic receptors that are highly expressed in the brain endothelium since it is assumed that such receptors have the capacity to convey these molecules across the BBB by the so-called receptor-mediated transcytosis (RMT) pathway. For the last two decades, the transferrin receptor (TfR) has been one of the major targets as it is one of the few endocytic receptors that is expressed more in brain capillary endothelial cells (ECs) than elsewhere in the vasculature. Other receptors, such as the insulin receptor and more recently the basigin (BSG) and the CD98 receptors [4], have been added to the list of possible targets for drug delivery to the brain. Despite extensive research, there is still a lack of understanding of the exact mechanism of RMT and the vesicular trafficking of different receptors in the brain endothelium. In vitro BBB models in combination with various techniques, such as permeability assays, microscopy, flow cytometry, ELISA, or Western blot, are often used to understand these mechanisms and other complex biological questions.

In the past few decades, various in vitro BBB models have been developed, including cell-based, non-cell-based, and stem cell-based models [5]. There is a growing appreciation for the development of stem cell-based BBB models using human embryonic and induced pluripotent stem cells (hiPSCs) as they represent the closest similarities to physiological human conditions [6]. Since the development of the first hiPSCs differentiation protocols [7,8], many protocol optimisation papers have been published [9,10,11,12,13,14,15,16,17]. Most protocols result in induced brain microvascular endothelial cells (iBMECs) that express BBB-specific transporters, efflux pumps, and key junctional proteins [7,8,9,10]. However, it has been reported that these cells also express epithelial-associated genes [16,18,19] and lack the expression of key adhesion molecules, such as ICAM-1, ICAM-2, and E-selectin, involved in immune cell migration across the BBB in vivo [15]. Furthermore, a recent study [16] demonstrated that iBMECs do not express many of the definitive transcripts that are essential for the development and maintenance of an endothelial fate, including KDR, vWF, ERGv, TAL1, CLDN5, SOX18, SOX17, ESAM, S1PR1, and PECAM1. A handful of protocols [15,16,17] have been developed to overcome epithelial characteristic issues of iBMECs. It is important to mention that iBMECs have been useful for the study of molecular transport and brain drug delivery as they express many key brain endothelial markers. They have, for example, been used to screen antibodies [20] and nuclear receptor ligands [21], to predict in vivo drug permeability [22,23], and to study antibody-triggered RMT [24].

Cell-based in vitro BBB models based on multiwell plates with microporous polymer membrane filters (e.g., Transwell^®^ or ThinCert^®^ inserts) are essential for high throughput screening and for assays that require many cells or large sample volumes. These assays include Western blot analysis, ELISA, simple fluorescence-based permeability assays, and microscopy in combination with immunocytochemistry to study transcellular localisation. However, the configuration of the system and the poor optical properties (mainly autofluorescence and light scattering), and the thickness of the track-etched membrane, especially in contact co-culture models, hinder the use of in situ imaging techniques. In situ microscopy, especially high-resolution live cell imaging, is an important technique to study cellular and spatiotemporal events, including intracellular trafficking and translocation, in real-time. The μSiM (microdevice featuring a silicon nitride membrane) platform [25,26], featuring ultrathin (~100 nm), highly permeable, and optically clear silicon nitride membranes, overcomes the aforementioned limitations of the conventional membrane-based culture systems. The µSiM has previously been used to set up various in vitro BBB models to study nanoparticle translocation with high-resolution in situ imaging [27] and immune cell trafficking [28,29]. The µSiM has been recently redesigned to enable a simple and rapid assembly from mass-produced components, eliminating the need for microfabrication tools [30]. Compared to the traditional hanging filter-based system, the flexible μSiM has a short distance from the objective to the cells, which, in combination with the ultra-thin membrane, allows high-resolution imaging in doubled layered co-culture setup.

The overall aim of our work was to study receptor-mediated antibody trafficking across contact iBMEC-astrocyte co-culture using high-resolution in situ imaging. To achieve this we set up a stem cell-based in vitro BBB model using commercially available iPS(IMR90)-4 cells on the µSiM device, a model we refer to as the µSiM-iBMEC. Testing several known iPSC differentiation protocols, we show the optimisation of iPS(IMR90)-4 derived iBMEC in vitro BBB model as monoculture and as contact co-culture with human foetal astrocytes (hAstros). The model was set up and characterised on both conventional Transwell and µSiM platforms. The iBMECs formed tight barriers, as measured by small molecule (sodium fluorescein (NaFl)) permeability, and expressed the main junctional proteins (such as Claudin-5, ZO-1, p120, and Occludin) and endothelial markers (vWF, MDR1, and GLUT1) on both platforms. The µSiM-iBMEC was then used to establish a co-culture model of the BBB to study the transcytosis of mAbs against the TfR and BSG receptors (15G11 hIgG1 [31] and BSG mAb#52 [32,33]) using high-resolution in situ imaging. Our results indicated that the antibodies accumulated in the iBMECs but not inside the hAstros over 24 h, indicating a tight BBB model. However, when the barrier was intentionally made leaky in control experiments with subconfluent iBMECs, antibodies accumulated inside both the iBMECs and hAstros.

## 2. Results

### 2.1. Differentiation Protocol Selection

We tested five iPS(IMR90)-4 differentiation protocols (Supplementary Material Figure S1) based on prior published protocols [9,10,12,14]. Differentiation protocols 1–3 consisted of four main steps. (1) First, the singularised iPS(IMR90)-4 cells were seeded onto Matrigel-coated plates. (2) To initiate the endothelial induction, the mTeSR (or mTeSR+) cell culture medium was switched to the unconditioned medium (UM) for six days. This step resulted in a mixed endothelial cell/neural progenitor cell culture. (3) On day 6, the UM was switched to an endothelial cell medium supplemented with retinoic acid (RA) and human fibroblast growth factors (hFGF) to selectively expand the endothelial cells (iBMEC specification step). (4) Finally, the iBMECs were purified by subculturing the cells on human collagen IV/fibronectin-coated surfaces. Protocol 4 had an additional step. Prior to the endothelial induction and specification step, the hiPSCs were treated with CHIR99021, a glycogen synthase kinase 3b (GSK-3b) inhibitor and canonical Wnt/β-catenin agonist, to direct hiPSCs to an intermediate mesoderm-like stage [10]. Furthermore, this protocol called for DeSR1 and DeSR2 medium instead of the UM (Figure S1). We did not manage to reproduce protocol 4, and although the differentiated cells looked endothelial-like prior to purification, they did not form tight barriers on collagen IV/fibronectin-coated filters (the measured transendothelial electrical resistance (TEER) was below 15 Ω·cm^2^ (Supplementary Material Figure S2)). Therefore, we decided to slightly modify protocol 4. In protocol 5, after the intermediate mesoderm-like stage, the cells were treated with the UM medium instead of the DeSR2 medium (Supplementary Material Figure S1). This differentiation protocol resulted in iBMECs with similar cell morphology to co-differentiation protocols 1 and 3 and relatively high TEER values (Supplementary Material Figure S2).

We noticed cell morphology changes (e.g., the cells became elongated, more fibroblast-like), cell detachment, and cell death during the endothelial induction step in case of protocols 1 and 2, i.e., when the initial hiPSCs seeding density was below 15,000 cells/cm^2^, (Supplementary Material Figure S3). These features were only noticed when the recommended 0.1 mM β-mercaptoethanol (β-ME) was used in the UM. Conversely, approx. ~0.05 mM β-ME (1:250,000 dilution of stock) did not have any harmful effect on the differentiation and resulted in iBMECs with the expected cell morphology. As a result of the high β-ME (0.1 mM) concentration and the relatively low initial hiPSC seeding density, protocol 2 (Figure S1) failed during the endothelial induction step. Only protocol 1 with lower β-ME concentration (~0.05 mM) and protocols 3–5 were further evaluated.

**Figure 1:**
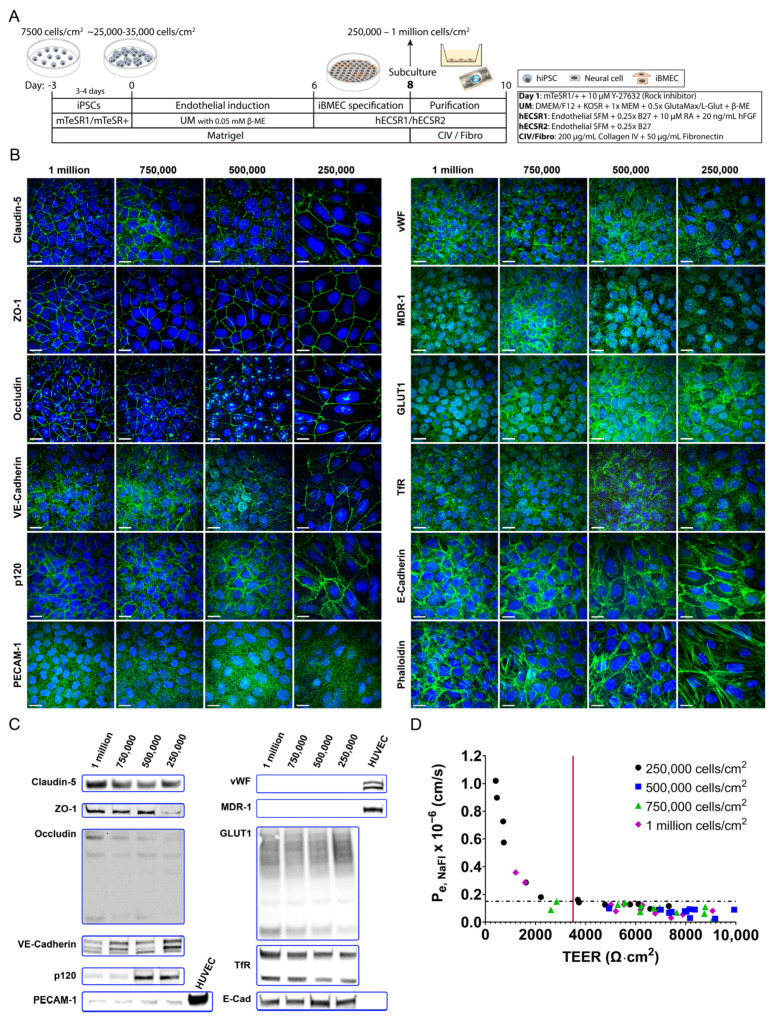
IMR90-4 hiPSC-derived iBMECs with different plating concentrations exhibit optimal barrier properties with some epithelial characteristics on ThinCert^®^ membranes. (**A**) Schematics of the final differentiation protocol for deriving iBMECs from IMR90-4 hiPSCs. The iBMEC-neuronal cell culture mix was subcultured on day 8 using four different plating densities, and the purified iBMECs were characterised or used on day 10 or day 11. (**B**) Representative maximum intensity projection (MIP) confocal images showing the expression of junctional proteins (Claudin-5, ZO-1, Occludin, VE-Cadherin, and p120), the lack/not correctly localised expression of PECAM-1 and endothelial markers (vWF, MDR-1 (P-glycoprotein 1) efflux transporter, GLUT1 glucose transporter, and TfR), E-Cadherin epithelial marker, and phalloidin. Markers: green, Hoechst 33,342 nuclei stain: blue. Scale bars represent 20 µm. 1 million, 750,000, 500,000, and 250,000 represent the plating densities in cells/cm^2^. (**C**) Western blot analysis shows the expression of endothelial markers. A slight expression of PECAM-1 and the lack of expression of vWF, MDR-1. (**D**) Relationship between paracellular permeability of sodium fluorescein (P_e,NaFl_) and TEER of IMR90-4 hiPSCs-derived endothelial cells (four or five independent experiments with triplicates). Cells were seeded on Greiner #665641 filters with four different seeding densities and were grown for two or three days. TEER was measured after the permeability experiment followed by 20 min equilibration at RT. The values are not temperature corrected and represent TEER at room temperature. Cells with TEER below 3500 Ω·cm^2^ (red line) represent cells with a not-intact barrier (visibly ruptured barrier in the case of 750,000 and 1 million cells/cm^2^ data points, non-confluent cell layer in the case of 250,000 cells/cm^2^ data points below ~800 Ω·cm^2^). The average overall TEER above 3500 Ω·cm^2^: 6924 (1492) Ω·cm^2^; 250,000: 5538 (1326) Ω·cm^2^, 500,000: 8010 (1326) Ω·cm^2^, 750,000: 7121 (1317) Ω·cm^2^, 1,000,000: 6673 (1283) Ω·cm^2^. With an intact cellular barrier, the effective permeability of NaFl was below 1.5 × 10^−7^ cm/sec.

In most of the hiPSC differentiation protocols, the cells are plated on Transwell^®^ or ThinCert^®^ filters at a seeding density of 1 million cells/cm^2^ [9,10,11,34], which results in a crowded cell population with cobblestone-like cell morphology; these iBMECs are much thicker and have smaller perimeters than typical primary endothelial cells in culture. Since our aim was to set up an in vitro hiPSC-based BBB model that would allow us to study transcellular trafficking using high-resolution imaging, the iBMECs’ plating density on collagen IV/fibronectin-coated filters needed to be optimised. Five different seeding densities, 1 million cells/cm^2^, 750,000 cells/cm^2^, 500,000 cells/cm^2^, 250,000 cells/cm^2^, and 125,000 cells/cm^2^, were tested and characterised on the Transwell^®^ platform. The barrier formation on the filters was assessed by TEER measurement and immunostaining for endothelial markers on days 10–12 (2 to 4 days on filter). The highest TEER values were measured on day 10, the TEER values only increased from day 10 to day 11 in cases of 250,000 cells/cm^2^ and 125,000 cells/cm^2^ plating densities (not shown). In the case of protocol 4 (based on the Qian et.al., 2017 protocol [10]), the cells looked endothelial-like during the iBMEC specification step, but not after the purification step. Furthermore, the iBMECs did not form a tight barrier on filters (TEER values were below 20 Ω·cm^2^) and did not express any of the tight junction proteins and endothelial markers shown in Supplementary Material Figures S4–S7. However, when UM was used instead of the recommended DeSR2 medium after the intermediate mesoderm-like stage (protocol 5), the cells reached a TEER of around 2000 Ω·cm^2^ at a seeding density of 1,000,000 cells/cm^2^. Only protocol 1 with low β-ME, protocol 3, and protocol 5 resulted in adequate TEER values and the immunocytochemical analysis indicated the expression of endothelial markers Claudin-5, VE-Cadherin, vWF, and MDR-1 (Figure 1, Supplementary Material Figures S4–S7). The lowest seeding density, 125,000 cells/cm^2^, resulted in a non-confluent monolayer and consequently a low TEER value. In general, the highest TEER was measured when the iBMECs were differentiated using protocol 1 (based on the Stebbins et. al., 2016 protocol [9]). These differentiated cells also expressed the markers shown in Supplementary Material Figures S4–S7; however, they are not included here as a broader panel of endothelial marker expression is presented in Figure 1. The highly porous (~12.57% porosity) 0.4 µm pore size Corning^®^ Transwell^®^ polycarbonate membranes (#3401) with translucent optical properties were used for the protocol optimisation studies. However, the Transwell^®^ #3401 membranes were switched to the clear and 0.4 µm pore size low porosity (0.25% porosity) polyester ThinCert^®^ membranes as the transparency of these membranes enabled us to monitor the cell attachment and growth after seeding with a common transmitted light microscope. The measured TEER values (using protocol 1) on these filters were superior to those grown on Transwell^®^ polycarbonate filters (Figure S2), which could be due to the surface properties and low porosity of the membrane or the cell attachment to the collagen IV/fibronectin-coated filters. Since the 125,000 cell/cm^2^ seeding density did not result in a confluent cell layer, this seeding density was discarded from further studies. The iBMEC barrier was characterised by the expression of junction protein complexes, endothelial markers, TEER measurement, NaFl permeability assay, and cell thickness measurement. The results presented in this study are based on iBMEC differentiation protocol 1 with 0.05 mM β-ME concentration (Figure 1A).

### 2.2. Effect of Seeding Density on the Barrier Properties

After the selection of the differentiation protocol (schematics in Figure 1A) and the filter material and format, we investigated the effect of initial filter plating density on BBB properties as well as the thickness of the iBMEC cell layer and the attached number of cells per surface area. Regardless of the initial cell seeding density, Claudin-5, ZO-1, Occludin, VE-Cadherin, and p120 catenin junctional proteins were expressed (Figure 1B,C, Supplementary Material Figures S8 and S9). Although the Western blot indicated a lower ZO-1 expression in iBMECs plated with 250,000 cells/cm^2^ density, the immunofluorescence (IF) signal was as strong as in the case of the other three seeding densities. Both Western blot and IF signals were stronger for p120 of 500,000 and 250,000 cells/cm^2^; however, it was still present in the iBMECs seeded with higher seeding densities. Endothelial markers von Willebrand factor (vWF), MDR-1, GLUT1, and TfR were also expressed by the cells (Figure 1B). The anti-vWF and anti-GLUT1 antibodies used for immunostaining also displayed a positive signal with cellular localisation according to the literature, but the negative results in Western blot studies on lysates from iBMECs call to question the specificity of the antibodies (Figure 1C and Figure S9). The PECAM-1 staining showed a nonspecific binding with incorrect subcellular antibody localisation (Figure 1B). Furthermore, the Western blot analysis results also indicated a very low expression of PECAM-1 in iBMECs, especially in comparison with HUVEC cells (Figure 1C and Figure S10). Although the differentiated iBMECs expressed typical endothelial markers and formed a tight barrier (TEER value is usually above 3000 Ω·cm^2^), they also showed some epithelial characteristics. Epithelial marker, E-Cadherin was expressed by iPS(IMR90)-4 derived iBMECs with high localisation on the plasma membrane (Figure 1B); however, not by HUVEC and hCMEC/D3 based on Western blot analysis (Figure 1C and Figure S10). As Figure 1B and Supplementary Material Figure S11 show, the F-actin localisation in iBMECs depended on the seeding density. Specifically, iBMECs with 250,000 cells/cm^2^ plating density had relatively high expression of stress fibers (inserted junctional stress fibers), whereas, at other seeding densities, the F-actin mainly accumulated at the plasma membrane and formed a cortical actin belt [35,36], and minimal stress fibers were present. F-actin expression on the plasma membrane overlapped with the junctional proteins (not shown), which is not surprising as in endothelial barriers, F-actin is anchored to adherens junction proteins [35].

The barrier tightness was further evaluated by measuring the paracellular permeability of NaFl (376.67 Da). Figure 1D shows the relationship between the effective permeability of NaFl and TEER. There is an exponential relationship between permeability and TEER. As Figure 1D shows, when the cells formed a tight barrier (i.e., the TEER is above 3500 Ω·cm^2^ after a 20 min equilibration period at room temperature), the NaFl permeability is below 1.5 × 10^−7^ cm/s. The average apparent and effective permeability values above the 3500 Ω·cm^2^ TEER threshold are listed in Table 1.

The lowest permeability (7.39 (2.47) × 10^−8^ cm/s) and highest average TEER (8010 (1326) Ω·cm^2^) were observed at a seeding density of 500,000 cells/cm^2^. The lowest TEER and highest permeability (5538 (1326) Ω·cm^2^ and 12.29 (2.22) × 10^−8^ cm/s) were given by iBMECs with 250,000 cells/cm^2^ filter plating density. It is worth noting that the permeability was generally measured on day 10, i.e., after 2 days on the filters; however, in the case of 250,000 cells/cm^2^, the cells were used on day 11 (after 3 days on filter) as the cells needed 3 days to form a tight barrier (Supplementary Material Figure S12). We observed these changes (longer culture time to achieve barrier tightness and lower attached cells density on filter) after we switched from mTeSR1 media to mTeSR+ media, which was used for hiPSC maintenance and during the first few days of differentiation (Figure 1A).

To investigate the effect of membrane material and porosity on the permeability, Corning^®^ Transwell^®^ polycarbonate filters with high porosity (~12.57% porosity, #3401) were used. The NaFl permeability through the empty filters and with cells (500,000 cells/cm^2^ seeding density) was significantly higher when the more porous filter was used (Table 2).

The effective permeability using high porosity Corning^®^ Transwell^®^ #3401 filters was approx. 1.31 (0.23) × 10^−7^ cm/s, confirming the formation of a tight barrier. This permeability value is approximately twice the value for cells grown on low porosity ThinCert^®^ polyester membrane. This observation is not surprising as the porosity, material, pore size, thickness, etc., of the membrane can be expected to affect the cell attachment, growth, and permeability. Furthermore, the different membrane structures and materials (Table 2) can be expected to impact the retention of fluorescent tracer molecules. Thus, the properties of the membrane should be reported; most of the studies in the literature are lacking this information, which could have an effect on the reproducibility of experiments and interpretation of permeability values.

### 2.3. Cell Thickness of iBMECs Grown on Transwell Platform

**Figure 2:**
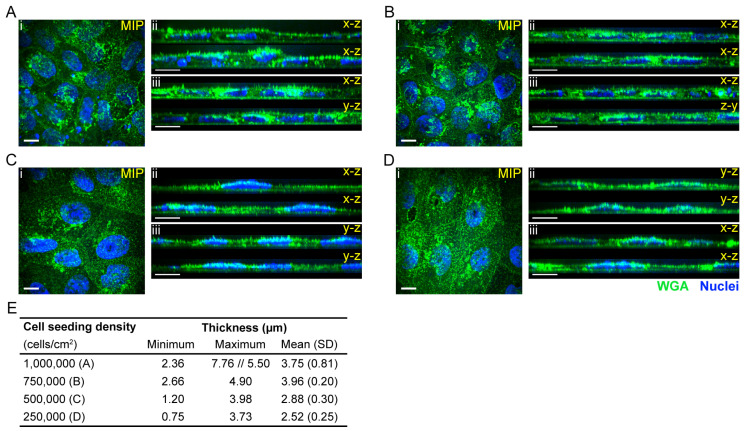
Lower iBMEC plating densities result in a monolayer with thinner, more elongated cells. The differentiated iBMECs were plated on ThinCert^®^ membranes at 1 million (**A**), 750,000 (**B**), 500,000 (**C**), and 250,000 (**D**) cells/cm^2^ densities. Representative maximum intensity projection (MIP) confocal micrographs of several *z*-stack in the middle section of the monolayer (**Ai**–**Di**) and the *x*–*z* (**Aii**–**Cii**) or *y*–*z* (**Dii**) cross-sectional view of two regions. (**Aiii**–**Diii**) Two cross-sectional images of other regions that were imaged (MIP is not presented). Scale bars represent 10 µm. (**E**) Minimum, maximum, and mean (SD) thickness of the iBMECs monolayer. The thickness was determined based on the cross-sectional view of several regions of several images.

To determine the cell thickness, the formaldehyde fixated iBMECs were stained with Alexa Fluor™ 488 conjugated wheat germ agglutinin (WGA) and imaged with 100× silicone objective. The average thickness was calculated based on the cross-sectional height of different regions of three images per seeding density. The higher seeding densities resulted in thicker, more compact monolayers. The attached cells had limited space to occupy, hence their shape was more cobblestone-like in the case of plating densities of 1 million and 750,000 cells/cm^2^ vs. 500,000 and 250,000 cells/cm^2^ (Figure 2A–D). The cross-sectional views also confirmed that in the cases of 1 million and 750,000 cells/cm^2^ seeding densities, the attached iBMECs formed a thicker, more packed monolayer and presented a more epithelial-like morphology (ii and iii in Figure 2A–D). The thickness of almost all iBMEC monolayers was below 5 µm; only regions with multiple layers had a thickness of ~7.8 µm (Figure 2E). The 1 million cells/cm^2^ plating density occasionally resulted in a barrier with regions with multiple cell layers. The average thickness of the iBMEC monolayer was 3.75 µm (1 million cells/cm^2^), 3.96 µm (750,000 cells/cm^2^), 2.88 µm (500,000 cells/cm^2^), and 2.52 µm (250,000 cells/cm^2^). Barriers with NaFl permeability below 1 × 10^−6^ cm/s are considered to be tight [9].

### 2.4. Seeding Efficiency on Transwell Platform–Number of Cells per Field of View vs. Plated Cells

To determine the seeding efficiency, the number of cell nuclei per field of view (FOV) was counted using the ImageJ cell counter plugin, then the attached cells/cm^2^ values were calculated. The number of attached iBMECs is shown in Table 3. In the case of 250,000 cells/cm^2^, approx. 57% of the plated cells attached to the surface, whereas only ~34% of the cells attached when the seeding density was 1 million cells/cm^2^. Hence, seeding more cells to the filters did not result in a higher cell attachment rate as the available space was limited and saturated around 750,000–1 million cells/cm^2^.

Based on the above results, the 500,000 cells/cm^2^ seeding density resulted in the tightest barrier (based on TEER and NaFl permeability measurements) with relatively large but thin cells that expressed most of the key endothelial markers with the exception of PECAM-1. Hence, this seeding density seems to mimic the in vivo morphology of brain endothelial cells and to be the best for non-microscopy-based experiments. However, for transcellular trafficking and co-localisation studies with confocal microscopy, we would recommend using a seeding density of 250,000 cells/cm^2^ with 2 or 3 days of cell growth due to the size and morphology of the attached iBMECs. Conventional confocal microscopes have a lateral resolution (*x*, *y*) of ~180–250 nm and an axial (*z*) resolution of ~500–700 nm [37]. Consequently, using larger and thicker cells provides the physical space needed to resolve the details of transcellular trafficking in microscopy. This means that iBMECs protocols resulting in cells with some epithelial-like characteristics provide a practical advantage for trafficking studies. Although a seeding density of 250,000 cells/cm^2^ did not give the tightest barrier (12.29 (2.22) × 10^−8^ cm/s vs. 7.39 (2.47) × 10^−8^ cm/s), barriers with NaFl permeability below 1 × 10^−6^ cm/s are considered tight for iBMECs [9].

### 2.5. Setting up the µSiM-iBMEC Model

**Figure 3:**
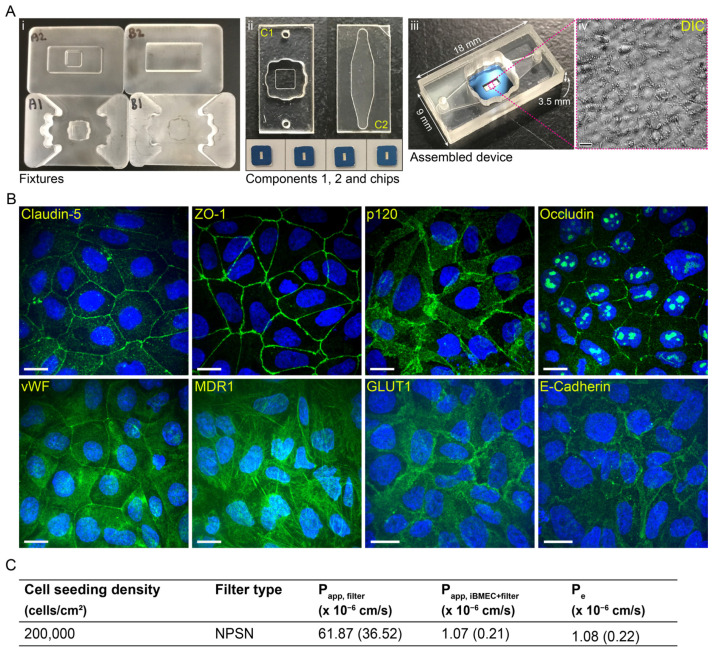
IMR90-4 hiPSC-derived iBMECs exhibit optimal barrier properties. (**A**) The m-µSIM device and its components. The device can be assembled with the guide of fixtures A and B (**i**) and is composed of an acrylic housing (component 1) that holds the nanoporous silicon nitride (NPSN) membrane chip with single-slot or dual-slot format and a bottom compartment (component 2) composed of a 150 µm thick pressure sensitive adhesive (PSA) channel and a 50 µm thick cyclic olefin copolymer (COP) bottom imaging layer (**ii**). The device (**iii**) can be easily assembled using the assembly kit and the differentiated iBMECs can be grown on the Transwell-style open well upper compartment. (**iv**) DIC image of the iBMCEs after 2 days on the membrane (scale bar: 20 µm). (**B**) Representative confocal micrographs showing the expression of junctional proteins (Claudin-5, ZO-1, p120, and Occludin) and endothelial markers (vWF, MDR1, and GLUT1), and E-Cadherin epithelial marker (green). Cell nuclei were stained with Hoechst 33342 (blue). Scale bars represent 20 µm. (**C**) Paracellular permeability of sodium fluorescein. Permeability (P) is presented as the mean (SD) of three independent experiments.

Next, the iBMEC BBB monoculture model was set up on the µSiM platform. The µSiM device and its components are illustrated in Figure 3A. Fixtures A1 and A2 were used to irreversibly bond the membrane chip and component 1 (acrylic housing with a Transwell-style open well and a pressure sensitive adhesive (PSA) sealing layer). Fixtures B1 and B2 were used to seal component 2 (bottom PSA channel with 150 µm heights and 50 µm thick cyclic olefin copolymer (COP) imaging layer) and component 1 with the membrane chip in it. In this study, we used a nanoporous silicon nitride (NPSN) membrane with 100 nm thickness, ~15% porosity, and ~50 nm pore size in both single-membrane [27,28] and double-membrane [30] chip formats. The single membrane chip format and the assembled µSiM are illustrated in Figure 3A(ii,iii).

As a first step, we investigated whether the previously found 500,000 and 250,000 cells/cm^2^ seeding densities were appropriate for culture on NPSN. Since the initial calculations only considered the surface area of the chip and not the entire top well (chip and surrounding sealing layer), the actual cell seeding densities were 400,000, 200,000, and 100,000 cells/cm^2^ instead of 500,000, 250,000, and 125,000 cells/cm^2^. As Supplementary Material Figure S13 shows, when the seeding density was low, i.e., 100,000 cells/cm^2^, the cells did not cover the entire membrane and failed to produce a confluent monolayer. We made the same observation when the cells were grown on the Transwell devices. Although the 400,000 iBMECs/cm^2^ seeding density resulted in a confluent monolayer with ZO-1 tight junction protein expression (not shown), the cell layer was found to be too compact to study intracellular trafficking when imaged with 60× water immersion objective (NA = 1.2). The mid-range seeding density, ~200,000 cells/cm^2^, similar to the Transwell platform, resulted in a confluent monolayer and large enough cells to resolve subcellular localisation during confocal imaging. Hence, this seeding density was used in further studies on the µSiM.

#### 2.5.1. Model Characterisation

The barrier properties of the µSiM-iBMEC model were evaluated first with immunocytochemistry. This analysis confirmed the expression of junctional proteins, Claudin-5, ZO-1, p120, and Occludin, as well as the endothelial markers vWF and MDR1, and the epithelial marker E-Cadherin (Figure 3B). Barrier tightness was further evaluated with the paracellular permeability measurement of NaFl. The measured permeability was 1.07 (0.22) × 10^−6^ cm/s, i.e., approximately one order of magnitude higher than it was for the IMR-90 cells grown on low porosity ThinCert^®^ filters (Figure 3C), but still ‘tight’ [9]. Due to a lack of compatible hardware, TEER measurements are not currently possible in the µSiM.

#### 2.5.2. Cell Count on µSiM

The number of nuclei per FOV was used to count the number of attached cells. Several FOVs of five independent differentiations were used for the calculations. The average attached cell count was 24.8 (2.4) cells/FOV, i.e., the attached cell density was 132,639 (12,662) cells/cm^2^. This means that ~66.3% of seeded cells attached to the membrane. While the attached cell density is a bit lower than it was for the 250,000 cells/cm^2^ density on the Transwell platform (132,639 vs. 142,730), ~10% (66.3% vs. 57.1%) more cells attached to the surface.

### 2.6. Trafficking of 15G11 hIgG1 and BSG mAb#52

**Figure 4:**
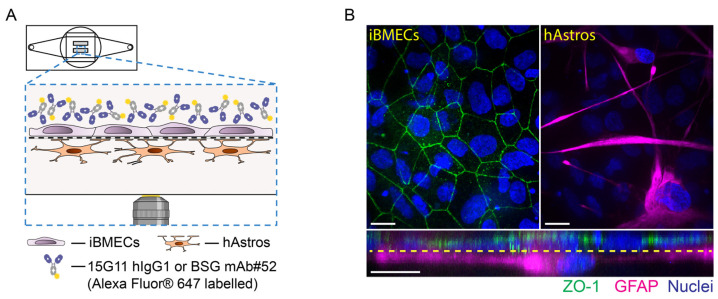
The µSiM-iBMEC contact co-culture model. (**A**) Schematic illustration of the µSiM-iBMEC contact co-culture model during the transcytosis experiment. (**B**) Immunocytochemical analysis of the μSiM-iBMEC co-culture model. Green: ZO-1 (iBMECs), magenta: GFAP (hAstros), blue: nuclei (both cell types); scale bars represent 20 µm.

Next, the µSiM-iBMEC model in combination with in situ imaging was used to investigate the endocytosis and transcytosis ability of two bivalent mAbs targeting the human TfR and BSG. The 15G11 hIgG1 [31] was used to follow TfR and BSG mAb#52 [32,33] was used to follow BSG. For these experiments, we used the µSiM-iBMEC contact co-culture model, in which the iBMECs were co-cultured with primary hAstros (Figure 4). Although the co-culture model was not characterised as thoroughly as the monoculture model, immunofluorescence staining confirmed the expression of junctional protein complexes in iBMECs, here illustrated with ZO-1 staining, and the glial fibrillary acidic protein (GFAP) in hAstros (Figure 4B).

**Figure 5:**
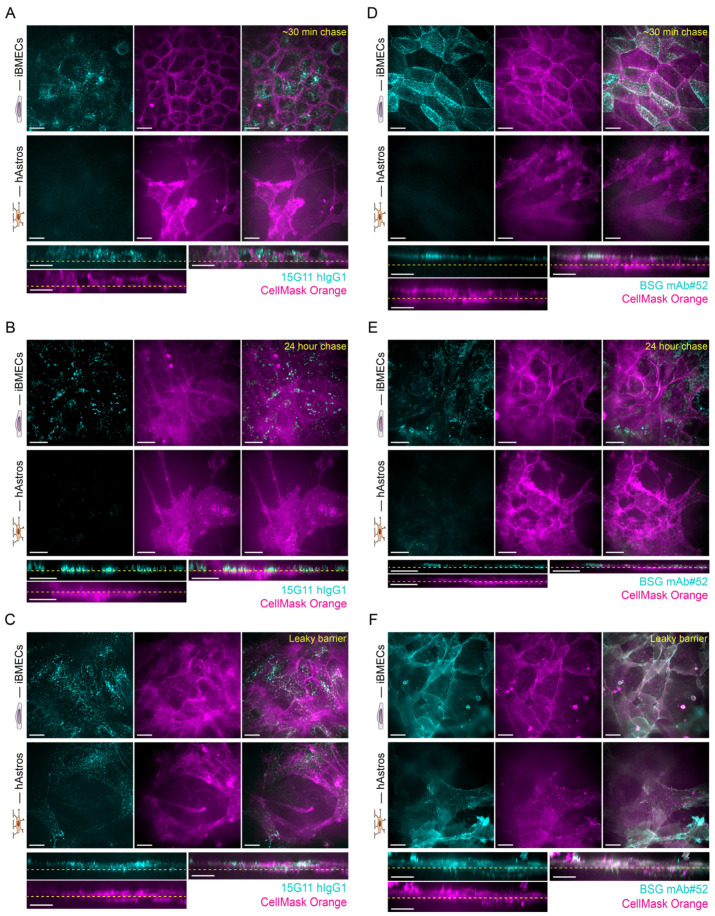
Transcytosis of Alexa Fluor 647 prelabelled high-affinity bivalent 15G11 hIgG1 and BSG mAb#52 across the µSiM-iBMEC model. Representative maximum intensity images and cross-sectional views showing the uptake of the 15G11 (**A**–**C**) and BSG mAb#52 (**D**–**F**) (cyan) in iBMECs and hAstros; the latter corresponds to the transcytosed mAbs. The apical side of the co-culture was exposed to Alexa Fluor 647 labelled 15G11 (500 nM) or BSG mAb#52 (100 nM) for 2 h or 10 min, respectively. The images represent the 30 min (**A**,**D**) and 24 h (**B**,**E**) chase times. Both iBMECs and hAstros were stained with 7.5 µg/mL CellMask Orange plasma membrane dye (magenta). The iBMECs formed a tight barrier in subfigures (**A,B,D**,**E**), whereas the barrier was leaky in subfigures (**C**,**F**). Scale bars represent 20 μm.

Molecular trafficking was studied by exposing the apical side of the µSiM-iBMEC co-culture to 500 nM Alexa Fluor 647 prelabelled 15G11 hIgG1 for 2 h or to 100 nM Alexa Fluor 647 prelabelled BSG mAb#52 for 10 min (pulse). After the 2 h or 10 min exposure times, the excess antibody solution was removed, and the cells were washed and imaged for up to 24 h (chase time). The iBMECs and hAstros were visualised by spinning disk confocal microscopy; both cell types were labelled by CellMask Orange plasma membrane stain. The experimental setup is illustrated in Figure 4A. Our results showed that up to the 24 h chase, both mAbs were internalised and accumulated in the iBMECs and not in the hAstros (Figure 5A,B,D,E) indicating that an insignificant amount of the mAbs was able to cross the tight iBMEC barrier. Conversely, when the iBMEC monolayer was visibly subconfluent, the mAbs accumulated inside both the iBMECs and hAstros (Figure 5C,F). While the subcellular localisation of the antibodies in the iBMECs was not studied in detail, results indicate that at early chase time points (30 min to 1 h) the BSG mAb#52 mainly accumulates on the cell surface, whereas at later times (after 24 h) the antibody is primarily localised in intracellular vesicles (Figure 5D,E). By contrast, the 15G11 already showed vesicular localisation at early chase times (Figure 5B).

## 3. Discussion

Macromolecules, especially mAbs, have potential for the treatment of neurodegenerative diseases [38,39]. However, the vesicular trafficking and the transcytosis mechanisms of targeted receptors are not fully understood. Here, we optimised a stem cell-based in vitro BBB model both on Transwell and µSiM platforms using iPS(IMR90)-4 cells that could be used to address these questions and to study the transcytosis and vesicular trafficking of both receptors and receptor ligands.

We tested several stem cell differentiation protocols, and among the tested protocols, a neural and endothelial co-differentiation protocol was selected and optimised for our studies. This protocol is a modified version of the first protocols of the Shusta Lab [7,8]; however, it was mainly based on some of the further optimised protocols [9,11,13]. These differentiation protocols lead to iBMECs that present both endothelial and epithelial characteristics [16,18,19]. A recent publication demonstrated the lack of canonical endothelial cell markers (CDH5, PECAM1, KDR (VEGFR2), APLNR, and eNOS) and critical ETS transcription factors (ETS1, ETV6, and FLI1) [16]. Furthermore, these iBMECs are not suitable for immune cell migration studies [15]. Our results also showed the expression of epithelial marker E-Cadherin (Figure 1 and Figure 3 and Supplementary Material Figure S10), and regardless of the differentiation protocols, PECAM-1 expression was almost never observed by immunostaining (Figure 1B and Supplementary Material Figures S4–S7). Although E-Cadherin is a typical epithelial marker, it has been found in brain endothelial cells as well [40]. The Western blot analysis only showed a low expression of PECAM-1 in iBMECs in comparison to endothelial cell lines HUVEC and hCMEC/D3, which could indicate either a low expression level in general or that only a very small population of the cells expressed PECAM-1 (Figure 1C and Supplementary Material Figure S10). Furthermore, the cell morphology and thickness (Figure 1A, Figure 2 and Figure 3B) of these cells resemble epithelial cells more than primary endothelial cells (thin and elongated cell morphology). The cell thickness of iBMECs can be advantageous as it enables the use of conventional confocal microscopy to study transcellular trafficking. Such studies are limited in in vitro models based on primary brain endothelial cells (BECs) as the thickness of these cells are below the axial resolution of the confocal microscope [41]. Higher resolutions with confocal microscopy can be achieved by the use of Zeiss’ Airyscan unit or Leica’s SP8 confocal microscope, or alternatively, the cells can be expanded after fixation (expansion microscopy [42]) and super-resolution can be achieved [41]. Moreover, these iBMECs expressed the major junctional complexes and endothelial markers, including efflux MDR-1 and influx transporter GLUT-1, (Figure 1 and Figure 3B), and formed a very tight barrier. The TEER value was above 3500 Ω·cm^2^ (average of 5538 (1326) Ω·cm^2^ for 250,000 cells/cm^2^ and 8010 (1326) Ω·cm^2^ for 500,000 cells/cm^2^ filter plating density) (Figure 1D, Table 1) and the paracellular permeability of NaFl was low (Figure 1D and Figure 3C, Table 1). Although the paracellular permeability of NaFl on the µSiM platform was one order of magnitude smaller than on ThinCert^®^ filters with similar seeding density (P_e,NaFl_: 1.08 (0.22) × 10^−6^ cm/s (200,000 cells/cm^2^, µSiM) vs. P_e,NaFl_: 1.23 (0.22) × 10^−7^ cm/s (250,000 cells/cm^2^, ThinCert^®^), see Figure 3C vs. Table 1), this value still indicated the formation of a tight barrier as it was statistically equivalent to the 1 × 10^−6^ cm/s threshold value [9]. Our data also demonstrated that permeability, especially apparent permeability, highly depends on the material and porosity of the membrane (Table 1 and Table 2). Based on the two-tailed unpaired *t*-test, there was a significant difference between the NaFl permeability (P_app,filter_) of low porosity ThinCert^®^ (#665641) and high porosity Corning^®^ Transwell^®^ #3401 filters (*p* < 0.0001). The effective permeability of cells grown on Corning^®^ Transwell^®^ #3401 filters was also significantly higher (based on the two-tailed unpaired *t*-test: *p* ≤ 0.0001, based on one-way ANOVA Tukey’s multiple comparison: *p* ≤ 0.001). The membrane is made from a different material and the porosity is 50 times higher, which could explain this result. In addition to the potential impact of membrane material and porosity on permeability measurements, the 100-fold greater thickness of conventional membranes means a ~100-fold greater surface area compared to µSiM membranes and therefore a much higher potential for molecular absorption to membrane surfaces in trafficking studies. Hence, the parameters of the membrane should always be reported as they might affect the in vitro permeability and transcytosis results.

In quiescent endothelium, F-actin forms a cortical rim or circumferential belt that is associated with tight junctions and adherens junctions [35,36,43]. Hence, our phalloidin staining indicates that iBMECs from a seeding density of 400,000 cells/cm^2^ (Figures S11 and S14) formed a tight and quiescent cell layer. Conversely, iBMECs at seeding densities of 200,000 and 250,000 cells/cm^2^ formed a confluent monolayer with non-distinctive cortical actin rim and stress fibers. Although the presence of stress fibers in endothelial cells can be an inflammatory response [35], we believe this was not the case as only cell number per cm^2^ and not medium and growth conditions were changed. The iBMECs at lower seeding densities were either not quiescent, or they presented a different endothelial F-actin organisation. The F-actin and stress fiber organisation seems to be very similar to the one present in human umbilical vein endothelial cells (HUVECs). In HUVECs, the stress fibers insert into the adherens junctions, linking the neighbouring endothelial cells together through the so-called discontinuous adherens junctions [44]. Discontinuous adherens junctions are highly dynamic and are elevated in response to tumour necrosis factor (TNF)-α [44].

After the model system was optimised and characterised on both Transwell and µSiM platforms, the compatibility of µSiM to study antibody trafficking was explored. For these studies, we selected TfR and BSG, which are two frequently targeted receptors for drug delivery across BBB. The iBMECs expressed both TfR and BSG receptors (Supplementary Material Figure S14), hence it was suitable for our studies. The TfR is one of the most investigated receptors in the brain regarding drug transport and numerous antibody constructs have been developed, such as bispecific antibodies [45,46,47], Brain Shuttle module [48], single-chain variable fragments [49], and transport vehicles [50,51,52]. BSG has not been studied as extensively as TfR; however, BSG-mediated uptake has been observed in mouse brain [4]. We also showed the transcytosis capability of four mAbs against BSG in vitro on porcine brain endothelial cells [33]. Here, we used bivalent 15G11 hIgG1 (Kd of approx. 5 nM [31]) and bivalent BSG mAb#52 [32,33] (Kd of 3 nM) to target TfR and BSG, respectively. Furthermore, B12 hIgG1 was used as isotype control (Supplementary Material Figure S16).

The chosen mAb concentrations and exposure times were determined by preliminary microscopy experiments in which different concentrations and exposure times were tested. The labelled 15G11 hIgG1 was relatively dim, therefore, a 500 nM concentration and 2 h exposure time were found to be optimal. At the same time, 100 nM concentration and 10 min exposure time of BSG mAB#52 already resulted in a strong signal with a low signal-to-noise ratio. Our results showed that none of the antibodies were able to cross the intact barrier. Instead, they accumulated inside the iBMECs even after a 24 h chase time (Figure 5). No antibody uptake was observed with B12 mAb confirming that our observations were not due to dye leakage; all antibodies were prelabelled with Alexa Fluor 647, which could potentially have caused some problems. Our findings are in agreement with other studies, which reported that bivalent antibodies with high binding affinity tend to accumulate in the endothelial cells, mainly inside the lysosomes, and BBB penetration is limited [45,48,49,53,54]. Consequently, many new studies use bispecific or monovalent antibodies or antibody fragment constructs with low or moderate affinities [47,48,49,50,51,52,53,54]. Although we have previously seen that BSG mAb#52 was able to cross the BBB in vitro [33], in that study, we used porcine brain endothelial cells (pBECs) and hAstros. The Western blot analysis data of our previous study also showed that BSG expression in hAstros is significantly higher than in pBECs [33], whereas the BSG expression in hAstros is significantly lower than in iBMECs (Figure S14), which could explain why the uptake in hAstros was insignificant in comparison to iBMECs. The expression of both TfR and BSG receptors was higher in iBMECs than in hAstros (Figures S14 and S15), which could also explain our results. The iBMECs and hAstros were imaged as a z-stack and since the membrane was only 100 nm thick, it was below the resolution of the microscope (z-stack resolution was 0.31 µm) and hence, there are stacks in which both iBMECs and hAstros are visualised. It is plausible that our spinning disk microscope was not sensitive enough to detect the mAb uptake in hAstros.

In this study, we presented an iPS(IMR90)-4 derived in vitro BBB models both on Transwell and µSiM platforms. Although our results indicated that both tested BSG mAb#52 and 15G11 hIgG1 do not seem to cross the intact µSiM-iBMEC barrier, we believe that the µSiM platform will be a valuable model system to understand receptor and receptor-ligand trafficking. This could be linked to an evaluation of optimised antibody formats targeting the TfR1 and BSG receptor and/or novel receptor targets expressed in the model.

## 4. Materials and Methods

### 4.1. The Modular µSiM Device

The m-µSIM device is composed of an acrylic top well fluidic access port to the underside channel (component 1), a nanomembrane chip with single-slot or dual-slot format, and a bottom compartment (component 2) composed of a 150 µm thick PSA channel and a 50 µm thick cyclic olefin copolymer bottom imaging layer. In this study, nanoporous silicon nitride (NPSN, SiMPore, NPSN100-1L, and NPSN100-2L) membranes were used (manufactured by SiMPore Inc., West Henrietta, NY, USA). The NPSN membranes had ~100 nm thickness, ~50 nm diameter pore size, and a porosity of ~15% (~10^8^ pores per mm^2^ [26]). Component 1 (top well) and component 2 (bottom channel) were manufactured at ALine Inc. (Signal Hill, CA, USA). The external surfaces of the components included an additional protective layer to maintain cleanliness and sterility until device assembly. The devices were assembled in-house in a sterile environment (a biosafety cabinet). First, the membrane chip with the flat side up (i.e., trench down) was placed on Fixture A1 (Figure 3A) using notched tweezers (Techni-Tool #758TW003, Worcester, PA, USA). The protective masks from both sides of Component 1 were removed with the help of straight-tipped tweezers (Techni-Tool #758TW534) before the component was placed over the chip, well-side down. Fixture A2 was pressed firmly onto Fixture A1 to bond the chip to Component 1. Component 2 was then removed from the protective strip and placed into Fixture B1 (exposed PSA and channel-side up). Component 1 with well-side up was then placed onto Component 2 and the two components were bonded by firmly pressing Fixture B2 onto Fixture B1. The assembled device was then removed from the fixture, and any air bubbles on the underside of the device were pressed out using straight-tipped and curved tweezers (Fine Science Tools #11003-12, Heidelberg, Germany). The assembled m-µSiM devices were further sterilised by UV light for 30 min to 1 h in the biosafety cabinet before using for cell culture. Step-by-step guides and tutorials can be found on the website: https://nanomembranes.org/modular-%c2%b5sim/ (accessed on 12 January 2023) and in a recent publication [30].

### 4.2. Cell Culturing

#### 4.2.1. Maintenance of hiPSC

Human iPS(IMR90)-4 (WiCell #WB65316, Madison, WI, USA) was maintained on Matrigel (Corning #356231 or #354277, Corning Inc., NY, USA; 8.7 µg/cm^2^ in DMEM/F12 (Sigma-Aldrich #D8437, Merck Life Science A/S, Søborg, Denmark))-coated 6-well plates in mTeSR1 or mTeSR Plus (mTeSR+) (STEMCELL™ Technologies #85850, #100-0276, Cambridge, UK), as previously described [9].

#### 4.2.2. Differentiation of hiPS(IMR90)-4 to iBMEC-like Cells (Final Differentiation Protocol)

Three days before initiating a differentiation, iPS(IMR90)-4 cells were washed once with Dulbecco′s Phosphate Buffered Saline (DPBS) (Sigma-Aldrich # D8537), dissociated with Accutase (Sigma-Aldrich #A6964, or STEMCELL™ Technologies #07920) for 3–5 min, collected by centrifugation (300× *g* for 5 min), and plated onto Matrigel-coated 6-well plates at a density of 7500 cells/cm^2^ in mTeSR1 or mTeSR1+ medium supplemented with 10 µM Rho-associated protein kinase (ROCK) inhibitor Y-27632 (Tocris Bioscience #1254; purchased from Fisher Scientific, Roskilde, Denmark). The hiPSCs were cultivated in mTeSR1 for three days or until they reached a density of 25,000–35,000 cells/cm^2^. A representative well was used to determine the cell density, by adding 1 mL of Accutase to the well for approx. 5 min; cell number was immediately counted (expected concentration: 250,000–350,000 cells/mL). To initiate the differentiation, mTeSR1 (or mTeSR1+) medium was switched to UM: 78.5% DMEM/F12 without L-glutamine (Thermo Fisher Scientific #21331020, Roskilde, Denmark), 20% KnockOut™-Serum Replacement, 1% MEM non-essential amino acid solution (Thermo Fisher Scientific #11140050), 0.5% (1 mM) GlutaMAX™ (Thermo Fisher Scientific #35050061) with ~0.05 mM (1:250,000 dilution of stock) β-mercaptoethanol (β-ME) (Sigma-Aldrich #M3148, Lot. #BCBV8182). The β-ME was added fresh every day, and the cells were cultured for an additional 5 days in UM. At day 6, the UM medium was switched to hECSR1: human endothelial serum-free medium (Thermo Fisher Scientific #11111044) supplemented with 20 ng/mL hFGF (R&D Systems #233-FB-025, Abingdon, UK), 10 mM RA (Sigma-Aldrich #R2625), and 0.5% or 0.25× (200× dilution of stock) B-27™ supplement (B27) (Thermo Fisher Scientific #17504001). After 2 days of culture in hECSR1 medium, i.e., on day 8, the cells were washed twice with DPBS and dissociated with Accutase (2 mL Accutase/well of a 6-well plate for 30–40 min at 37 °C) and plated in hECSR1 medium onto collagen IV/fibronectin pre-coated membranes, where they were grown for 2 to 3 days (iBMEC purification step). The human placenta-derived collagen IV (Sigma-Aldrich #C5533) was used at a concentration of 400 µg/mL, whereas the human plasma-derived fibronectin (R&D Systems #1918-FN or Corning #11533610) was used at 100 µg/mL in the collagen IV/fibronectin mixture. On day 8, the dissociated iBMEC-neuronal cell mixture was used directly or cryopreserved in hECSR medium supplemented with 30% FBS and 10% DMSO for later use.

#### 4.2.3. Maintenance of Primary Human Astrocytes

Commercially available human brain progenitor-derived astrocytes (Thermo Fisher Scientific #N7805-100) were cultured in Dulbecco’s Modified Eagle Medium (DMEM) (Thermo Fisher Scientific #31966-021) supplemented with 1% N-2 Supplement (Thermo Fisher Scientific #17502-048), 10% fetal bovine serum (FBS) (Sigma-Aldrich #F9665), and 20 ng/mL EGF (Thermo Fisher Scientific #PHG0314), referred as astrocyte medium. The cells were cultured on Matrigel (8.7 µg/cm^2^ in DMEM/F12)- or Geltrex (Thermo Fisher Scientific #A1413302; 13.5 µg/cm^2^ in DMEM)-coated cell cultured plastics and used between passages 1 and 5. Cells were passaged using Accutase^®^ and seeded in a density of 10,000 cells per cm^2^. Cells were cultured in an incubator at 37 °C with 5% CO_2_/95% air and saturated humidity. The astrocyte medium was changed every two days.

#### 4.2.4. iBMEC Monoculture on Transwell System

On day 8, the collagen IV/fibronectin-coated transparent polyester ThinCert^®^ inserts (12-well format, 1.131 cm^2^, 0.4 µm pore size, 2 × 10^6^ pores/cm^2^, i.e., approx. 0.25%, porosity, Greiner Bio-One GmbH #665641; purchased from In Vitro A/S, Fredensborg, Denmark) were seeded with iBMECs at four different seeding densities (250,000; 500,000; 750,000, and 1,000,000 cells/cm^2^). On day 9, the medium was changed to hECSR2 (hECSR1 without RA or hFGF) and the cells were used for the experiment on day 10 or 11 in case the TEER was not sufficiently high on day 10. It should be noted that during the differentiation protocol optimisation, Corning^®^ Transwell^®^ polycarbonate membrane inserts (12-well format, 1.12 cm^2^, 0.4 µm pore size, 1 × 10^8^ pores/cm^2^, i.e., approx. 12.57%, porosity, Corning #3401) were used.

#### 4.2.5. iBMEC Monoculture and Co-Culture on µSiM

For monoculture experiments, the differentiated IMR90 cells were plated at a density of 200,000 cells/cm^2^ (100,000 and 400,000 cells/cm^2^ were also tested) to the top reservoir of UV light sterilised m-µSiM devices with collagen IV/fibronectin-coated NPSN membrane. The next day (day 9), the hECSR1 medium was changed to hECSR2. The cells were used for experiments on day 10. For the transcytosis experiments, the iBMECs were co-cultured with hAstros. In this case, both sides of the membrane were pre-coated with collagen IV/fibronectin, and the hAstros (20 µL of 650,000–900,000 cells/mL cell suspension in astrocyte medium, i.e., approx. 15,000–20,000 cells/cm^2^) were seeded to the bottom reservoir 1–2 days prior to iBMEC seeding; the top reservoir was filled with astrocyte medium. After cell seeding, the devices (parafilm-covered) were immediately flipped upside down to allow the cell settlement to the bottom side of the membrane. They were flipped back to the original orientation after approximately 1.5 h incubation at 37 °C and were cultured for an additional 1–2 days with daily medium change. Prior to iBMEC seeding, the astrocyte medium was changed to hECSR1 in both reservoirs. The iBMECs were seeded at a density of 200,000 cells/cm^2^ and were grown for 2 days as described above. When day 8 cryopreserved iBMECs were used, the hECSR1 media was supplemented with 10 µM ROCK inhibitor [55]. Due to the small channel reservoir, the µSIM devices were kept in a 6 cm cell culture dish (Greiner Bio-One GmbH # 628160) or in a well of a 6-well plate with sterile MilliQ water-wetted Kimcare wipes (Kimberly-Clark #7552, Kleenex wipes #7432, Kimberly-Clark Nordic, Bagsværd, Denmark) that provided enough humidity, and the channels did not dry out.

### 4.3. Characterisation of iBMEC Monoculture or Co-Culture Models

#### 4.3.1. Immunocytochemistry

The iPS(IMR90)-4 cells were differentiated and subcultured on Transwell^®^ or ThinCert^®^ filters or m-µSiM devices as described above. Two or three days after subculturing, the cells were washed with pre-heated HBSS or hECSR2 medium and were fixed with 4% formaldehyde (Sigma-Aldrich #441244) for 15 min at room temperature or with 100% methanol for 5 min at −20 °C, followed by three PBS washes. The cells were permeabilised with 0.1% Triton X-100 (Sigma-Aldrich #X100) (PBS-TX) in PBS for 10 min and blocked in 2% bovine serum albumin (BSA) (VWR #0332, Søborg, Denmark) (in 0.05% PBS-TX) for 30 min. After that, the polyester or polycarbonate membranes were cut out from the inserts into up to four pieces. Cells were then incubated with primary antibodies (Table S1) for 1 h at room temperature. After incubation, cells were washed three times for 5 min with PBS and incubated with fluorescently labelled secondary antibodies (Table S1) for 1 h in dark at room temperature, followed by rinsing three times for 5 min with PBS. Both primary and secondary antibodies were diluted in 2% BSA in 0.05% PBS-TX. The secondary antibody solution also contained 0.6 µg/mL Hoechst 33342 (Sigma-Aldrich #B2261) and in certain cases, Alexa Fluor™ 568 conjugated Phalloidin (only formaldehyde-fixated cells). The Transwell^®^ or ThinCert^®^ membranes were mounted in fluorescence mounting medium (Dako #S3023, AH diagnostics A/S, Tilst, Denmark) on microscope glass slides (Hounisen Laboratorieudstyr A/S #2510.1205BL, Skanderborg, Denmark) and covered by 12 or 13 mm #1.5 coverslips (Thermo Fisher Scientific #NA, VWR #631-1578). As negative controls, the cells were only stained with secondary antibodies.

#### 4.3.2. Transendothelial Electrical Resistance (TEER)

TEER values were measured using an EVOM Epithelial Volt/Ohm meter (World Precision Instruments, Friedberg, Germany) and ’chopstick’ electrodes (Greiner ThinCert^®^ inserts) or Endohm chamber system (Corning^®^ Transwell^®^ filters). The TEER was measured on day 10 (2 days on filter) and the TEER of a cell-free collagen IV/fibronectin-coated filter was used as blank. To calculate the TEER, the blank corrected EVOM readout in Ω was multiplied by the surface area of the filter (1.12 cm^2^ for Corning, 1.131 cm^2^ for Greiner filter), i.e., the TEER value is [Ω·cm^2^]. The TEER value changed rapidly during the measurement as a drop in temperature can artificially increase TEER. However, after a 20 min equilibration at room temperature, the measured values were stable, and it was possible to compare the batch-to-batch variation of the values. Since it was not possible to measure the values at 37 °C, it was measured at RT after a 20 min equilibration, which resulted in a more stable TEER value that did not fluctuate.

#### 4.3.3. Paracellular Permeability of NaFl

##### Transwell System

The paracellular permeability of 10 µM NaFl (376.27 Da) (Sigma-Aldrich #F6377-100G) was measured to determine the tightness of the iBMECs. On day 8, the differentiated iBMECs were subcultured onto collagen IV/fibronectin-coated ThinCert^®^ inserts at a density of 1 million, 750,000, 500,000, and 250,000 cells/cm^2^. The NaFl permeability was typically assessed on day 10 or day 11. On the day of the experiment, the cells were washed once with HBSS to remove the dead cell layer, and fresh hECSR2 medium was added to both apical and basolateral sides (700 µL into the top chamber and 1500 µL at the bottom chamber). To initiate the paracellular transport, 70 µL hECSR2 medium was removed from the apical well and 70 µL 100 µM NaFl working solution was added to the upper well. Immediately after this, 150 µL medium was removed from the bottom chamber and placed into a 96-well plate, and 150 µL fresh hECSR2 was added back to the bottom chamber (time 0). Then, the cells were placed on an orbital shaker at 100 rpm rotation in a 37 °C incubator. Sample from the basolateral side was collected at 15, 30, 45, and 60 min time points. At the last time point, an additional 150 µL was collected from the top chamber and transferred to a 96-well plate. Furthermore, 150 µL of hECSR2 medium with and without 10 µM NaFl was added in three wells of the 96-well plate and used for background subtraction. The fluorescence intensity of the sample was read by a CLARIOstar^®^ Plus microplate reader (BMG Labtech, Ortenberg, Germany) (λ_Exc_ = 485 nm, λ_Em_ = 515 nm, Bandwidth = 15 and 20 nm, respectively), and the paracellular permeability was calculated. The permeability coefficient was determined by using the clearance principle [56]. First, the clearance volume at each time point was calculated.

(1) $$Clearance\;volume=\frac{V_{B}\cdot B_{t}}{T_{60min}}$$

where V_B_ is the volume of the bottom chamber (1500 µL); B_t_ is the corrected relative fluorescence unit (RFU) of the bottom chamber at time, t; T_60min_ is the RFU of the top chamber at 60 min (this value is assumed to be constant as it remains largely the same). Then, the clearance volume vs. time was plotted and the permeability was calculated using the slope of the linear regression line for both the culture and the blank filter. The following equations were used:

(2) $$P_{app}\left[\frac{cm}{s}\right]=\frac{m}{A\cdot 60}$$

where P_app_ is the apparent permeability, m is the slope, A is the cross-section area of the membrane [cm^2^], and 60 is used as a conversion from minute to second. The final endothelial permeability (P_e_) was calculated based on Equation (2):

(3) $$\frac{1}{P_{e}}=\frac{1}{P_{app,iBMECs+filter}}-\frac{1}{P_{app,filter}}$$

where P_app,iBMECs+filter_ is the apparent permeability of the iBMEC monolayer (cells + filter), and P_app,filter_ is the apparent permeability of the collagen IV/fibronectin-coated blank porous membrane. The permeability experiments were repeated three times in technical triplicates; presenting the data as mean (SD).

##### The m-µSiM

The paracellular permeability of 10 µM NaFl across the m-µSiM-iBMEC model was measured slightly differently. The iBMECs were cultured and grown on m-µ-SiM as described above, and the permeability measurement was conducted on day 10. The experiment was initiated by removing 50 µL hECSR2 medium from the apical well (100 µL volume in total) and immediately adding 50 µL 20 µM NaFl working solution to the well. Due to the small volume of the basolateral channel, only the sampling was done only once. At the 60 min time point, a 5 µL sample was removed from the basolateral side using a p10 pipette and loaded into a well of a 384-well plate (PerkinElmer #6008280, Perkinelmer Danmark A/S, Skovlunde, Denmark). The fluorescence intensity was read by a CLARIOstar^®^ Plus microplate reader and the paracellular permeability was calculated similarly as for the Transwell systems. However, here, the apparent permeability was calculated slightly differently, only using a single time point:

(4) $$P_{app}\left[\frac{cm}{s}\right]=\frac{B}{T}\frac{V_{B}}{A\cdot t\cdot 60}$$

where P_app_ is the apparent permeability, B is the RFU of the sample at 60 min time point, T is the RFU of 10 µM NaFl (i.e., top chamber concentration at time 0), V_B_ is the volume of the bottom channel (0.01 mL), A is the cross-section area of the membrane (0.014 cm^2^ or 0.028 cm^2^ for single-slot or dual-slot format, respectively), and t is the time (60 min). The permeability values are presented as the mean (SD) of three independent experiments.

#### 4.3.4. Western Blotting

The iBMECs were cultured on Transwells at different seeding densities for two days as described above, whereas the hAstros were cultured either in a Matrigel-coated T25 flask or a 6-well plate until 80–90% confluency. The cells were washed with ice-cold PBS and lysed with ExB lysis buffer (composed of 150 mM NaCl, 2 mM MgCl_2_, 2 mM CaCl_2_, 10 mM HEPES, 1% Triton X-100, and cOmplete protease inhibitor cocktail (Roche Diagnostics GmbH #11697498001, Basel, Switzerland)). The protein concentration of the cell lysates was determined by the BCA assay. A total of 5 µg of protein was loaded and resolved on 4–12% bis-tris ExpressPlus™ PAGE gels (GenScript Biotech #M41215, Rijswijk, The Netherlands) and transferred to nitrocellulose membranes via iBlot 2 dry blotting system (Thermo Fisher Scientific #IB21001). The membranes were blocked in 0.1% Tween 20/tris-buffered saline (TBST) with 5% skim milk (Sigma-Aldrich #70166) for 1 h at RT and were incubated with primary antibodies (see Table S1) overnight at 4°C. The membranes were then washed five times with TBST with 1% skim milk and incubated with secondary antibodies (see Table S1) for 1 h. The membranes were washed five times with TBST and imaged using iBright 1500 (Thermo Fisher Scientific). The images were further processed with iBright Analysis Software (version 5.0, Thermo Fisher Scientific Inc.) or Adobe Photoshop.

#### 4.3.5. Cell Attachment Efficiency

The immunofluorescence micrographs stained for nuclei iBMECs cultured of ThinCert^®^ or m-µSiM platforms were used to determine the number of attached cells. The counting was based on the number of nuclei/FOV counts. The nuclei were counted manually using the Cell counter plugin of ImageJ/Fiji (version 2.3.1). The field-of-view (FOV) area was calculated as follows: each image comprised 512 × 512 pixels, i.e., one FOV could be calculated as (voxel size × 512)^2^. In the case of the 60× objective, the *x*–*y* voxel size is 0.267 µm, i.e., one FOV is approx. 1.9 × 10^−4^ cm^2^. The number of nuclei/FOV or the number of nuclei/cm^2^ is reported in the paper. The data had a normal distribution; hence, it is presented as mean (SD).

#### 4.3.6. The Thickness of the iBMEC Monolayer

The thickness of the iBMEC monolayer grown for two or three days on ThinCert^®^ filters was determined based on 3D confocal micrographs. After formaldehyde fixation, the cells were stained with 5 µg/mL Alexa Fluor 488 conjugated WGA for 10 min. After that, the cells were washed three times with DPBS, permeabilised with 0.1% Triton X-100, blocked with 2% BSA in 0.05% PBS-TX, and stained with primary and secondary antibodies and Hoechst as described above. The mounted cells were imaged with a spinning disk confocal microscope and a 100× silicone objective. The images were reconstructed using Arivis Vision 4D software (version 3.4, Arivis AG, Rostock, Germany) and the thickness of several different regions and images were measured as the height of the cross-section views.

### 4.4. Transcytosis Experiments

For transcytosis experiments, the iBMECs were co-cultured with hAstros on the µ-SiM as described above. On the day of the experiment, the hESCR2 cell culture medium was removed, the cells were washed one or two times to remove dead cells, and fresh medium was added. Approx. 1 h after the medium change, the apical surface of the iBMECs was exposed to 100 nM Alexa 647 labelled BSG#52 mAb for 10 min or 500 nM Alexa 647 labelled 15G11 hIgG1 for 2 h. After the 10 min or 2 h pulse, the excess BSG#52 mAb or 15G11 was removed, the cells were washed once with hECSR2, and both apical and basal sides were stained with 7.5 µg/mL CellMask™ Orange plasma membrane stain (Thermo Fisher Scientific #C10045) for 5 min. The imaging of both iBMECs and hAstros layers was done with spinning disk confocal microscopy at 30 min to 1 h and 24 h chase times (“z-stacks” of several regions). Alexa 647-labelled B12 hIgG1 (provided by H. Lundbeck A/S) at a concentration of 500 nM was used as a negative control. The cells were incubated with the B12 hIgG1 for 2 h and were imaged at 24 h chase time.

The labelled 15G11 hIgG1 was provided by H. Lundbeck A/S, whereas the BSG#52 mAb and B12 hIgG1 were labelled in-house using the Alexa 647 protein labelling kit (Alexa Fluor™ 647 Protein Labeling Kit; ThermoFisher Scientific, Invitrogen #A20173). The labelling was done according to the manufacturer’s instructions, and the protein concentration was measured with NanoDrop.

### 4.5. Image Acquisition, Image Analysis

All confocal images were acquired using a spinning disk confocal microscopy system consisting of a CSU-X1 spinning disk unit (Yokogawa Electric Corporation, Tokyo, Japan) and an Andor iXon-Ultra 897 EMCCD camera (Andor, UK) or a Hamamatsu ORCA-Fusion BT C15440-20U sCMOS camera (Hamamatsu Photonics, Hamamatsu City, Japan), mounted on an inverted fully motorised Olympus IX83 microscope body and a UPlanSApo 60x/NA1.20 (WD = 0.28 mm) water immersion objective or a UPlanSApo 100xS/NA1.35 (WD = 0.20 mm) silicone immersion objectives (Olympus Corporation, Tokyo, Japan). The z-step size was 0.26 µm for the 100× objective and 0.28 µm or 0.31 µm for the 60× objective. The images were acquired by Olympus cellSens software (version 3.1.1, Olympus, Hamburg, Germany) and were processed using Imaris (version 8.2.1, Bitplane AG, Schlieren, Switzerland) and Arivis Vision 4D (version 3.3, 3.4, 3.5). The confocal micrographs are presented as maximum intensity XY projection over the entire z-stack or several z-slices or cross-sectional views (stated in the figure legend). The size of the scale bar is stated in the figure legends.

The following excitation laser lines and emission filters were used: Alexa Fluor 647 conjugated antibodies and Abberior STAR RED-coupled secondary antibodies: λ_ex_ = 640 nm, λ_em_ = 680/42 nm bandpass filter; Alexa Fluor 568 or Atto 550-coupled secondary antibodies, Alexa Fluor 568 coupled Phalloidin and CellMask™ Orange: λ_ex_ = 561 nm, λ_em_ = 625/90 nm bandpass filter, Alexa Fluor 488-coupled secondary antibodies and WGA: λ_ex_ = 488 nm, λ_em_ = 525/50 nm bandpass filter, and nuclei (Hoechst 33342): λ_ex_ = 405 nm, λ_em_ = 440/521/607/700 nm quad-band bandpass filter. The autofluorescence of the ThinCert^®^ polyester filter was strong in the 405 nuclei channel (see Supplementary Material Figure S17), therefore, the captured signal was corrected using TOP-HAT background correction using a custom Python script in Arivis Vision 4D software. The TOP-HAT Python scripts are available in the Supplementary Material File S1; the object size, channel, and folder locations were modified and kept constant within the image sets.

### 4.6. Statistics

The normality of the permeability, TEER, and all experimental data was tested and confirmed with GraphPad Prism 9.0.2 (GraphPad Software, Inc., San Diego, CA, USA) using its built-in normality tests and QQ plots (not shown). Since the experimental data exhibited a normal Gaussian distribution, they are presented as mean (SD).

## Figures and Tables

**Table 1:** Paracellular permeability of sodium fluorescein. Permeability (P) is presented as the mean (SD) of at least three independent experiments with triplicates.

Cell Seeding Density (Cells/cm^2^)	P_app,IMR90+F_ (×10^−8^ cm/s)	P_e_ (×10^−8^ cm/s)	TEER (SD) (Ω·cm^2^)
1,000,000 ^a^	8.24 (3.22)	8.36 (3.30)	6673 (1283)
750,000 ^a^	9.16 (3.35)	9.39 (3.43)	7121 (1317)
500,000 ^a^	7.18 (2.35)	7.39 (2.47)	8010 (1326)
250,000 ^a^	12.07 (2.14)	12.29 (2.22)	5538 (1326)
500,000 ^b^	13.10 (2.31)	13.14 (2.32)	5102 (1412)

Cells were grown on ^a^ Greiner Bio-One #665641 or ^b^ Corning #3401 filters. P_app,IMR90+F_—apparent permeability through IMR90 derived endothelial cells grown on filter, P_e_—“real” endothelial permeability. Membrane specifications: polyethylene terephthalate membrane with 10 µm thickness, 0.4 µm pore size, with 2 × 10^6^ pores/cm^2^, i.e., 0.25% porosity (Greiner Bio-One #665641); polycarbonate membrane with 10 µm thickness, 0.4 µm pore size, with 1 × 10^8^ pores/cm^2^, i.e., 12.57% porosity (Corning #3401). P_app,Greiner filter_: 683.76 (115.51) × 10^−8^ and P_app,Corning filter_: 5368.24 (756.11) × 10^−8^ cm/s.

**Table 2:** Specifications of ThinCert^®^ and Transwell^®^ membranes and the apparent permeability of sodium fluorescein across these membranes.

	Membrane Specifications						P_app,F_
Cat. No	Format	Type	Thickness	Pore Size	Porosity	Optical Prop.	(×10^−6^ cm/s)
Greiner Bio-One #665641	12-well	PET	10 µm	0.4 µm	2 × 10^6^ pores/cm^2^ 0.25% porosity	Transparent	6.84 (1.16)
Corning #3460	12-well	PET	10 µm	0.4 µm	4 × 10^6^ pores/cm^2^ 0.50% porosity	Transparent	23.03 (4.27)
Corning #3401	12-well	PC	10 µm	0.4 µm	1 × 10^8^ pores/cm^2^ 12.57% porosity	Translucent	53.68 (7.56)

PC: polycarbonate, PET: polyethylene terephthalate.

**Table 3:** Number of cells on filters on day 2 or day 3 (250,000 cells/cm^2^). Values are presented as mean (SD) of at least four independent iBMEC differentiations. A minimum of 10 images (usually >30 images) per differentiation were used for calculating the number (no.) of attached cells.

Cell Seeding Density (Cells/cm^2^)	No. of Cells/FOV	No. of Cells/cm^2^	Attached Cells (% of Plated)
1,000,000	63.9 (14.2)	341,766 (76,155)	34.2
750,000	58.6 (15.2)	313,393 (81,165)	41.8
500,000	41.6 (17.0)	222,582 (90,715)	44.5
250,000	26.7 (7.6)	142,730 (40,831)	57.1

FOV—field of view, Number of differentiations used: 4 (1 million cells/cm^2^) or 5 (all others).

## Data Availability

The datasets generated and/or analysed during the current study are available from the corresponding author upon reasonable request.

## Supplementary Materials

The following supporting information can be downloaded at: https://www.mdpi.com/article/10.3390/ijms24065624/s1. The File S1 is the TOP-HAT Python scripts that were used for background correction.
