# Supplementary material for: Modelling a Human Blood-Brain Barrier Co-Culture Using an Ultrathin Silicon Nitride Membrane-Based Microfluidic Device
Source: Int J Mol Sci. 2023 Mar 15;24(6):5624. doi: 10.3390/ijms24065624 (PMC10058651; doi:10.3390/ijms24065624)
Supplement: Supplementary file 1 [file ijms-24-05624-s001.zip › Supporting Materials-Table S1 & Figures S1-S17.pdf]

SUPPORTING MATERIALS

Modelling a Human Blood-Brain Barrier Co-Culture Using an Ultrathin Silicon Nitride Membrane-Based Microfluidic Device

Diana Hudecz [1], Molly C. McCloskey [2], Sandra Vergo [3], Søren Christensen [3], James L. McGrath [2], Morten S. Nielsen [1,*]

[1] Department of Biomedicine, Aarhus University, 8000 Aarhus, Denmark
[2] Department of Biomedical Engineering, University of Rochester, Rochester, NY 14627, USA
[3] Biotherapeutic Discovery, H. Lundbeck A/S, 2Valby, 2500 Copenhagen, Denmark
[*] Correspondence: mn@biomed.au.dk

Table S1. List of antibodies used in immunocytochemistry studies and Western blot analysis and cellular markers.

Antigen	Antibody type	Manufacturer, Cat. no	Concentration	
			IF	WB
Primary antibodies				
Claudin-5	Mouse monoclonal	Thermo Fisher Scientific, 35-2500	2.5 µg/mL	0.5 µg/mL
Claudin-5 - Lu	Rabbit polyclonal	Provided by H. Lundbeck A/S	4.4 µg/mL	N/A
E-Cadherin	Mouse monoclonal	BD Biosciences, 610181	5 µg/mL	0.25 µg/mL
GFAP (GA5)	Mouse monoclonal	Thermo Fisher Scientific, 14-9892-82	5 µg/mL	0.5 µg/mL
GLUT1	Rabbit polyclonal	Provided by H. Lundbeck A/S	5.0 µg/mL	1.0 µg/mL
MDR-1	Rabbit polyclonal	Provided by H. Lundbeck A/S	4.5 µg/mL	0.9 µg/mL
Occludin	Rabbit polyclonal	Thermo Fisher Scientific, 71-1500	2.5 µg/mL	1.5 µg/mL
p120 catenin	Mouse polyclonal	BD Biosciences, 610133	2.5 µg/mL	0.25 µg/mL
PECAM-1 (Lu) (2H8)	Hamster monoclonal	Provided by H. Lundbeck A/S	10 µg/mL	N/A
PECAM-1/CD31	Mouse monoclonal	R&D systems, BBA7	8 µg/mL	1.0 µg/mL
TfR	Rabbit polyclonal	Provided by H. Lundbeck A/S	7.0 µg/mL	0.7 µg/mL
VE-Cadherin	Rabbit polyclonal	Provided by H. Lundbeck A/S	5.5 µg/mL	1.1 µg/mL
vWF	Rabbit polyclonal	Provided by H. Lundbeck A/S	5.5 µg/mL	1.1 µg/mL
ZO-1	Mouse monoclonal	Thermo Fisher Scientific, 61-7300	2.5 µg/mL	1.0 µg/mL
Secondary antibodies				
Anti-Human IgG	Goat, HRP-coupled	Millipore, AP112P	N/A	1:5000
Anti-Mouse IgG	Horse, HRP-coupled	Cell Signaling, 7076s	N/A	1:2000
Anti-Mouse IgG	Goat polyclonal, Alexa Fluor 488-coupled	Thermo Fisher Scientific, A11029	2 µg/mL	N/A
Anti-Mouse IgG	Goat polyclonal, Alexa Fluor 647-coupled	Thermo Fisher Scientific, A21235	2 µg/mL	N/A
Anti-Mouse IgG	Abberior STAR RED	Abberior, STRED-1001-500UG	1 µg/mL	N/A
Anti-Rabbit IgG	Goat, HRP-coupled	Cell Signaling, 7074s	N/A	1:2000
Anti-Rabbit IgG	Goat polyclonal, Alexa Fluor 488-coupled	Thermo Fisher Scientific, A11034	2 µg/mL	N/A
Anti-Rabbit IgG	Goat polyclonal, Alexa Fluor 647-coupled	Thermo Fisher Scientific, A21244	2 µg/mL	N/A
**Other cellular markers**		**Manufacturer, Cat. no**	**Concentration**	
CellMask™ Orange		Thermo Fisher Scientific, C10045	7.5 µg/mL	
Hoechst 33342		Sigma, B2261	0.6 µg/ml	
Phalloidin (Alexa Fluor™ 568)		Thermo Fisher Scientific, A12380	1:40 (5 U/mL)	
WGA, Alexa Fluor™ 488 Conjugate		Thermo Fisher Scientific, W11261	5 µg/ml	

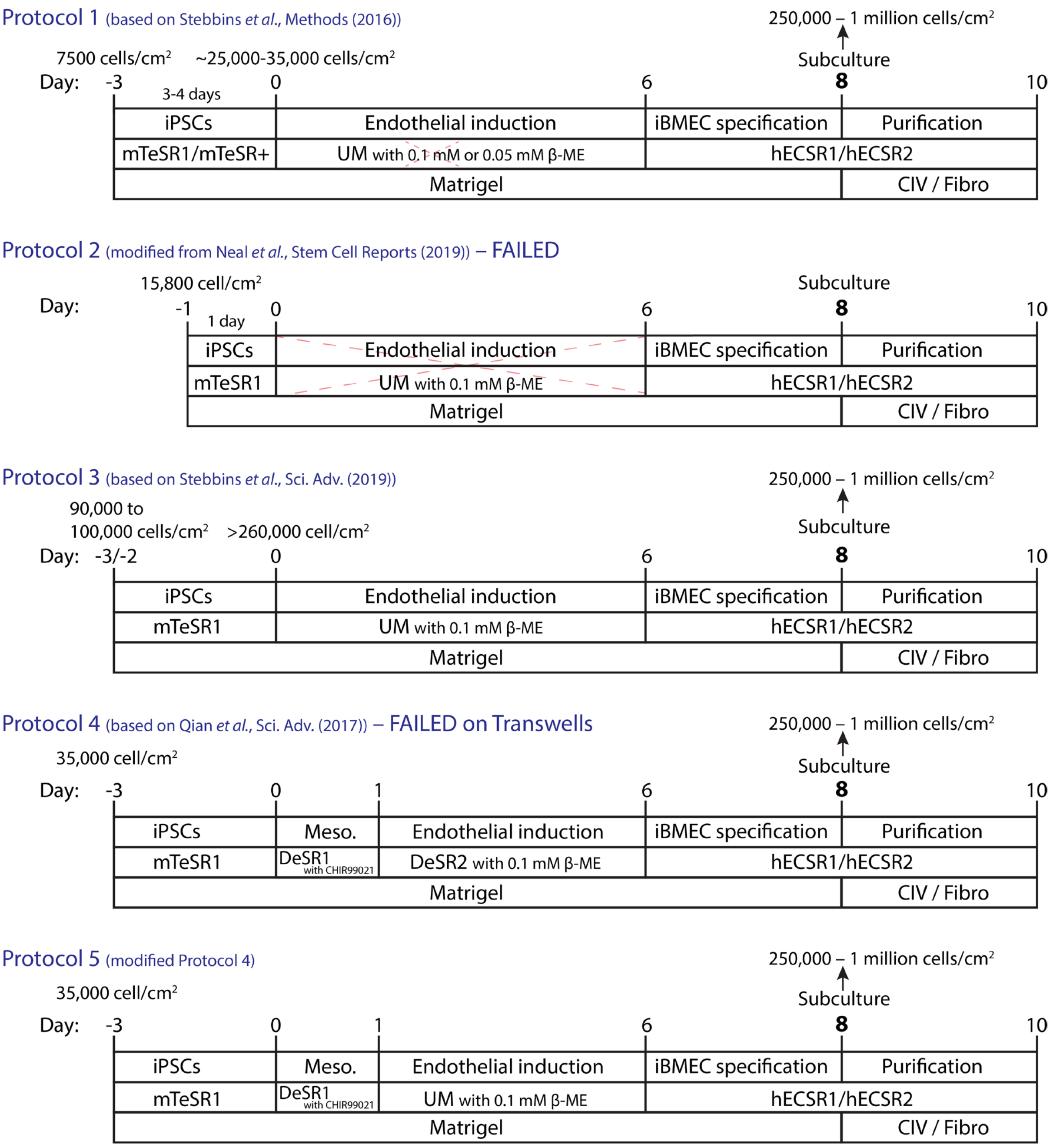

Day 1: mTeSR1/+ + 10 μM Y-27632 (Rock inhibitor)
UM: DMEM/F12 + KOSR + 1x MEM + 0.5x GlutaMax/L-Glut + β-ME
hECSR1: Endothelial SFM + 0.25x B27 + 10 μM RA + 20 ng/mL hFGF
hECSR2: Endothelial SFM + 0.25x B27
DeSR1: DMEM/F12 + 1x MEM-NEAA + 0.5x GlutaMax + 0.1 mM β-ME
DeSR2: DeSR1 + 1xB27
CIV/Fibro: 200 μg/mL Collagen IV + 50 μg/mL Fibronectin

Figure S1. Schematics of the tested differentiation protocols for deriving iBMECs from IMR90-4 hiPSCs. The protocols were based on the following publications: Stebbins *et al.*, Methods (2016) [1], Neal *et al.*, Stem Cell Reports (2019) [2], Stebbins *et al.*, Sci. Adv. (2019) [3], and Qian *et al.*, Sci. Adv. (2017) [4]. In protocols 1 and 3, B27 supplement was used instead of the recommended platelet-poor plasma-derived serum was replaced with.

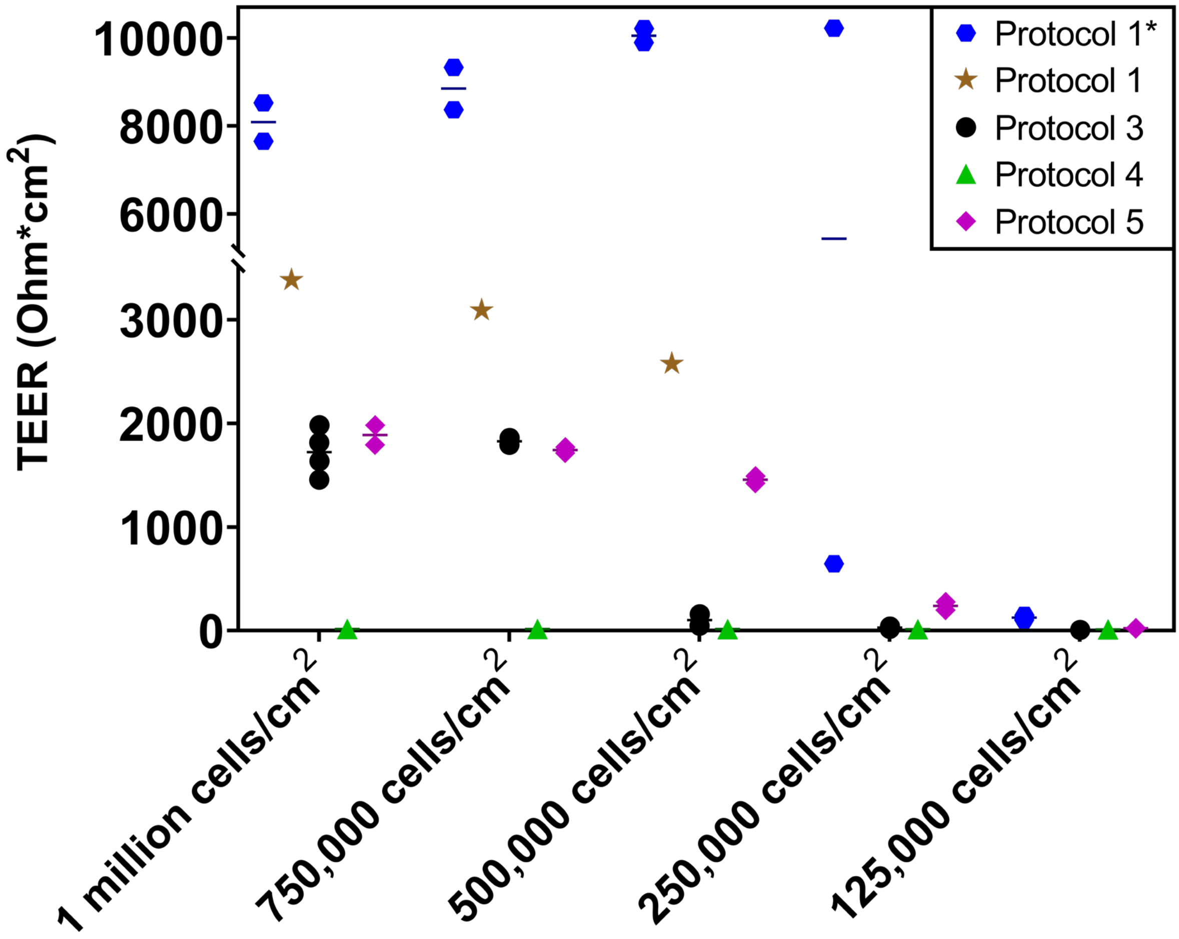

Figure S2. TEER on day 2 on the filter (day 10 differentiation). Cells were grown on Corning® Transwell® high porosity polycarbonate filters (#3401) (Protocol 1-5) or on Greiner ThinCert® low porosity polyester membrane (#665641) (Protocol 1*).

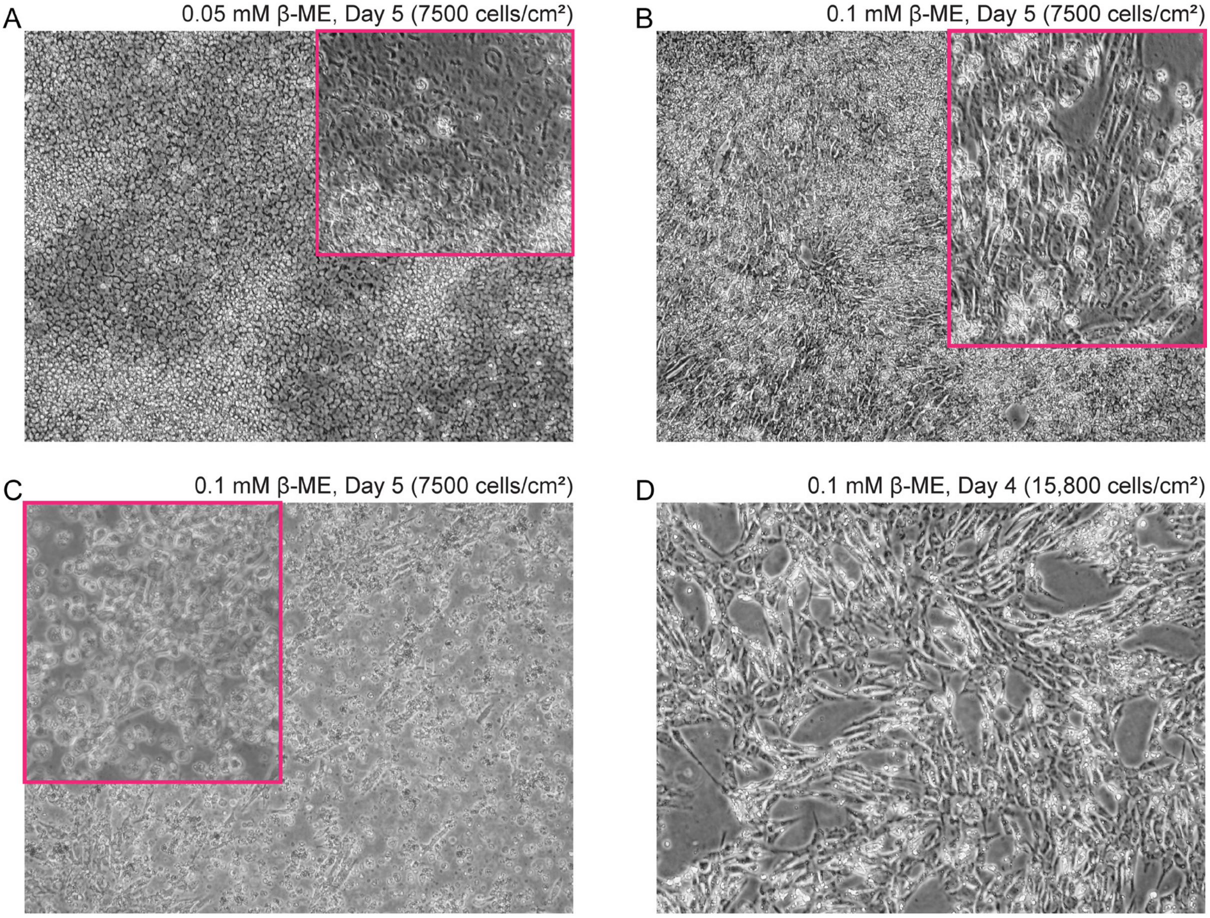

Figure S3. Effect of 0.1 mM β-mercaptoethanol (β-ME) on cell morphology during differentiation of IMR90-4 hiPSCs. Morphology changes were observed during the endothelial induction step (UM supplemented with 0.1 mM β-ME) when the initial plating density was below ~15,000 cells/cm^2, *ie.*, during protocol 1 **(A-C)** and protocol 3 **(D)**.

Protocol 3, 2 days on filter (Day 10), MeOH fixation

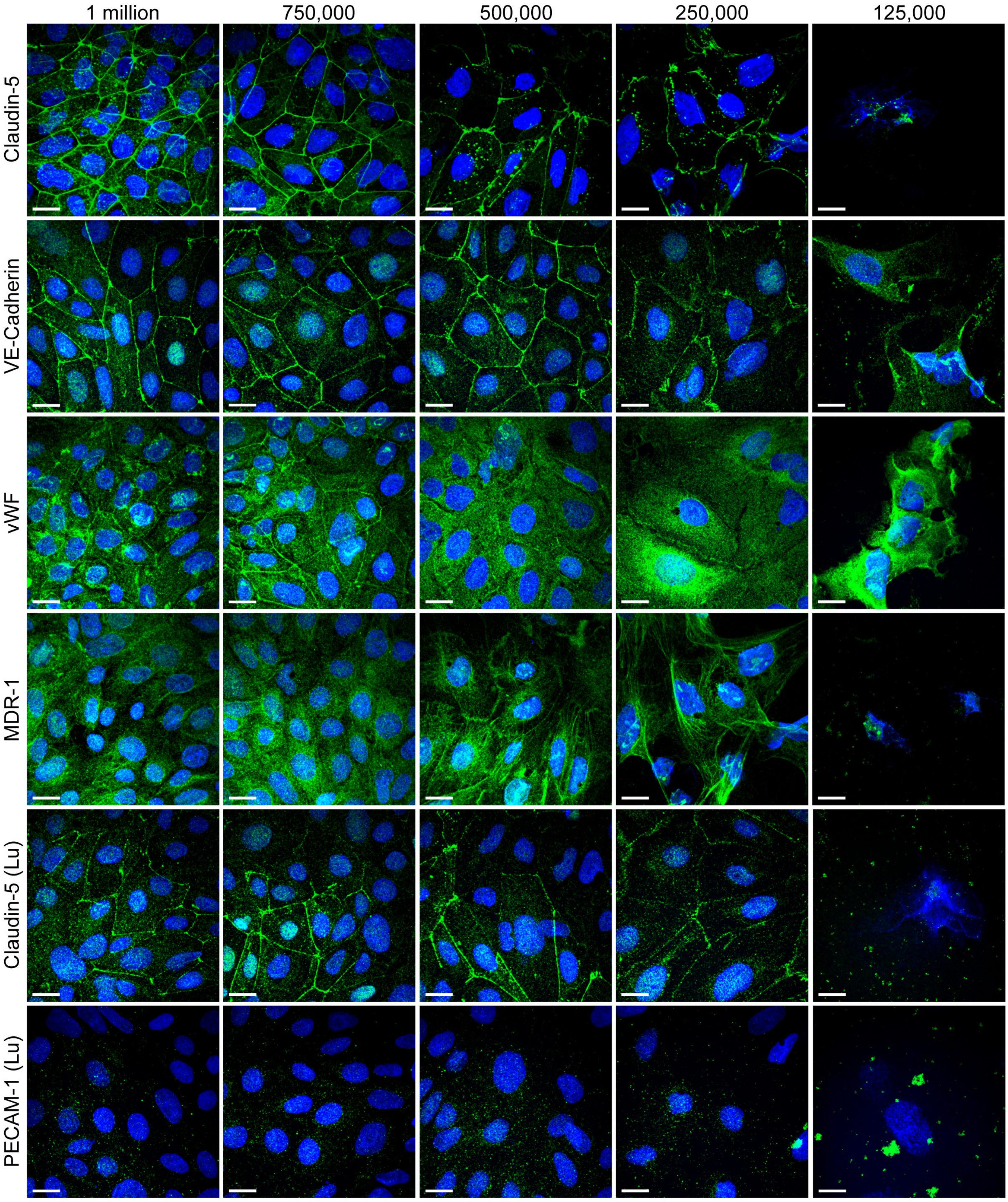

Figure S4. Immunocytochemical characterization of iBMECs derived from IMR90-4 hiPSCs using protocol 3. Representative maximum intensity projection (MIP) confocal images showing the expression of junctional proteins (Claudin-5, VE-Cadherin, and PECAM-1) and endothelial markers (vWF, MDR-1). The iBMECs were grown on Corning® Transwell® polycarbonate filters (#3401) for 2 days and fixated with MeOH. Markers: green, Hoechst 33342 nuclei stain: blue. Scale bars represent 20 µm. 1 million, 750,000, 500,000, and 250,000 represent the plating densities in cells/cm^2.

Protocol 3, 4 days on filter (Day 12), 4% PFA fixation

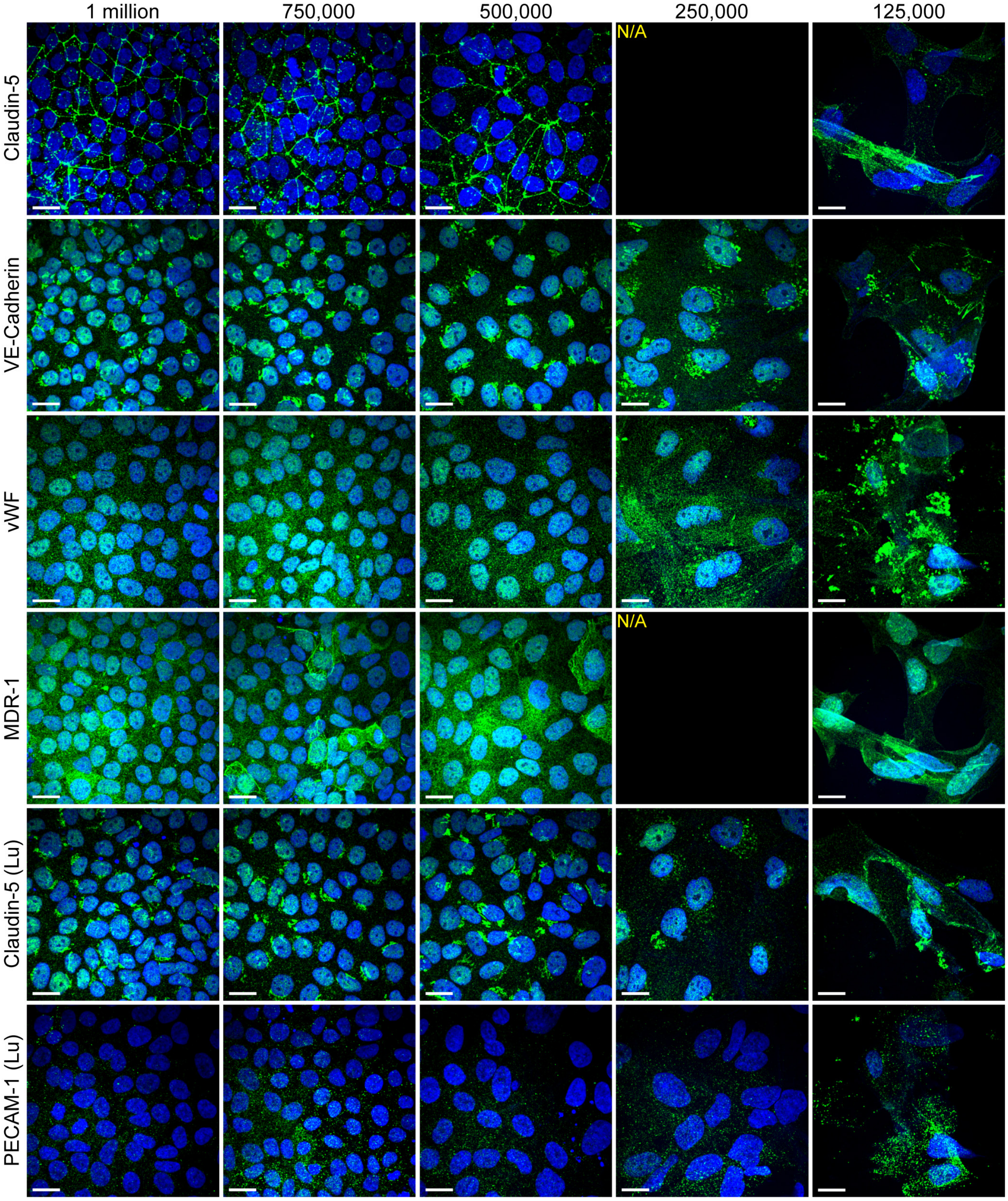

Figure S5. Immunocytochemical characterization of iBMECs derived from IMR90-4 hiPSCs using protocol 3. Representative MIP confocal images showing the expression of junctional proteins (Claudin-5, VE-Cadherin, and PECAM-1) and endothelial markers (vWF, MDR-1). The iBMECs were grown on Corning® Transwell® polycarbonate filters (#3401) for 4 days and fixated with 4% formaldehyde solution (4% PFA). Markers: green, Hoechst 33342 nuclei stain: blue. Scale bars represent 20 µm. 1 million, 750,000, 500,000, and 250,000 represent the plating densities in cells/cm^2.

Protocol 5, 2 days on filter (Day 10), MeOH fixation

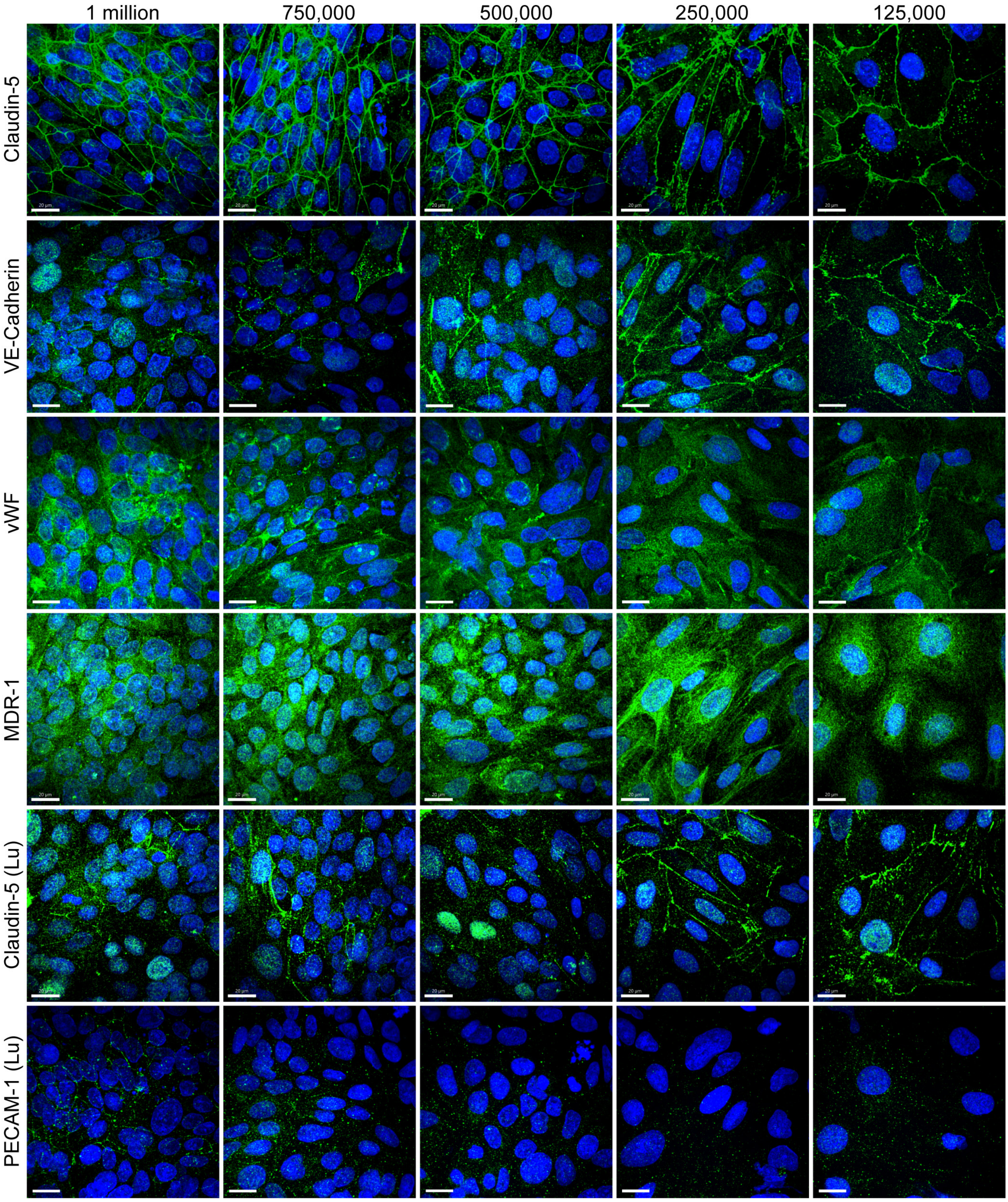

Figure S6. Immunocytochemical characterization of iBMECs derived from IMR90-4 hiPSCs using protocol 5. Representative MIP confocal images showing the expression of junctional proteins (Claudin-5, VE-Cadherin, and PECAM-1) and endothelial markers (vWF, MDR-1). The iBMECs were grown on Corning® Transwell® polycarbonate filters (#3401) for 2 days and fixated with MeOH. Markers: green, Hoechst 33342 nuclei stain: blue. Scale bars represent 20 µm. 1 million, 750,000, 500,000, and 250,000 represent the plating densities in cells/cm^2.

Protocol 5, 4 days on filter (Day 12), 4%PFA fixation

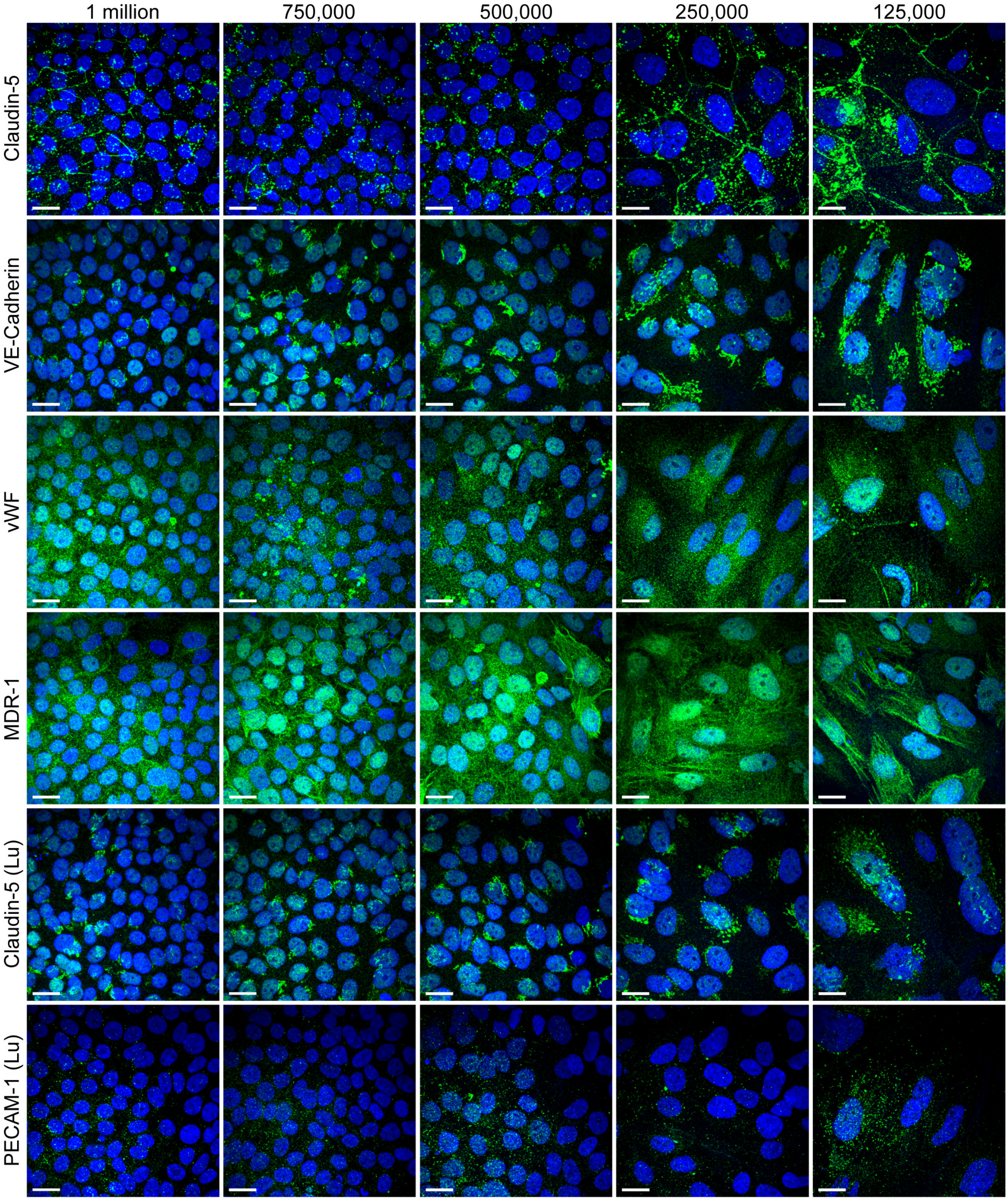

Figure S7. Immunocytochemical characterization of iBMECs derived from IMR90-4 hiPSCs using protocol 5. Representative MIP confocal images showing the expression of junctional proteins (Claudin-5, VE-Cadherin, and PECAM-1) and endothelial markers (vWF, MDR-1). The iBMECs were grown on Corning® Transwell® polycarbonate filters (#3401) for 4 days and fixated with 4% formaldehyde solution (4% PFA). Markers: green, Hoechst 33342 nuclei stain: blue. Scale bars represent 20 µm. 1 million, 750,000, 500,000, and 250,000 represent the plating densities in cells/cm^2.

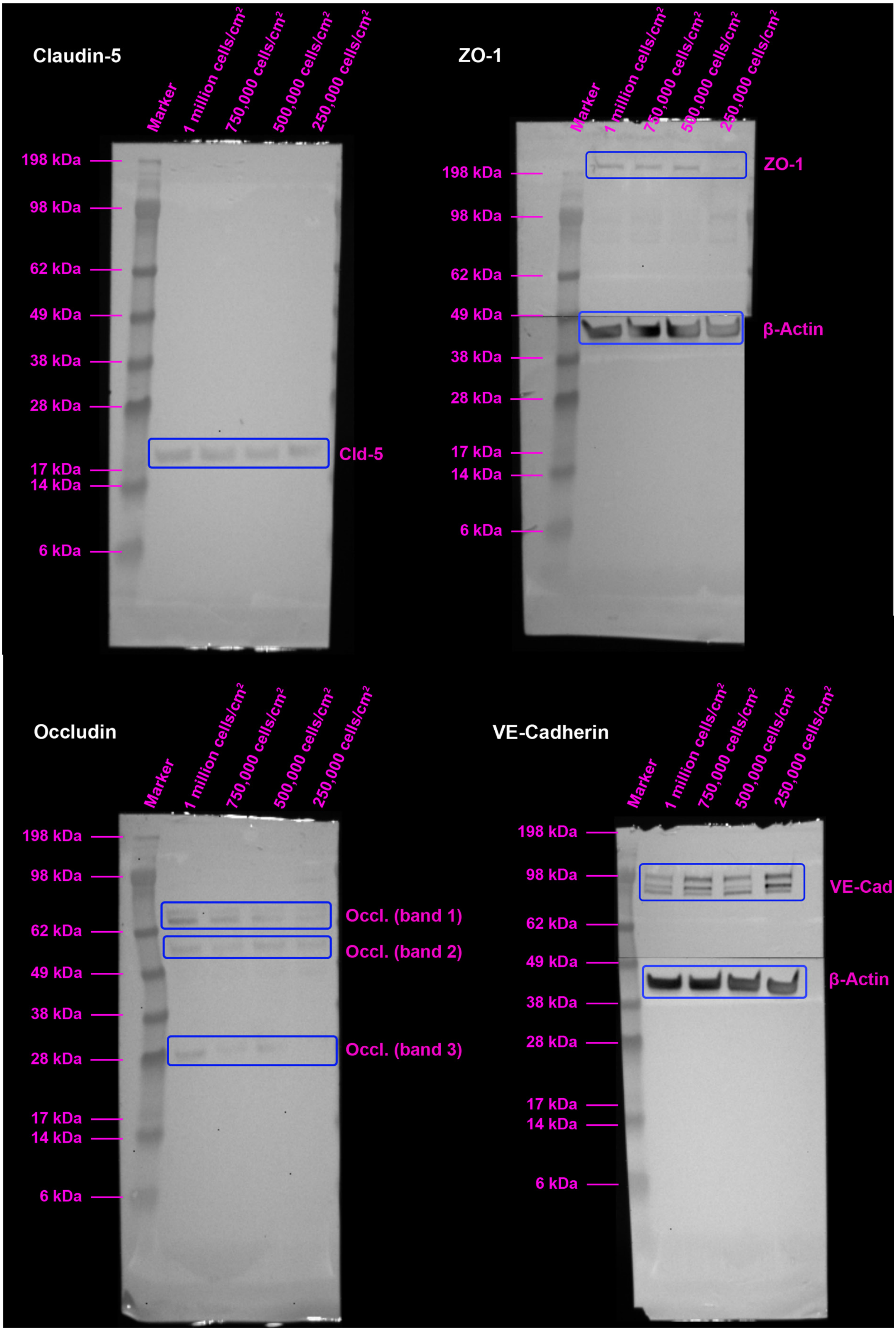

Figure S8. Extended Western blot results of Figure 1 (part 1). Western blot analysis shows the expression of junctional proteins in IMR90-4 hiPSC-derived iBMECs with different plating concentrations (cells were grown on low porosity ThinCert® (#665641) filters for two days).

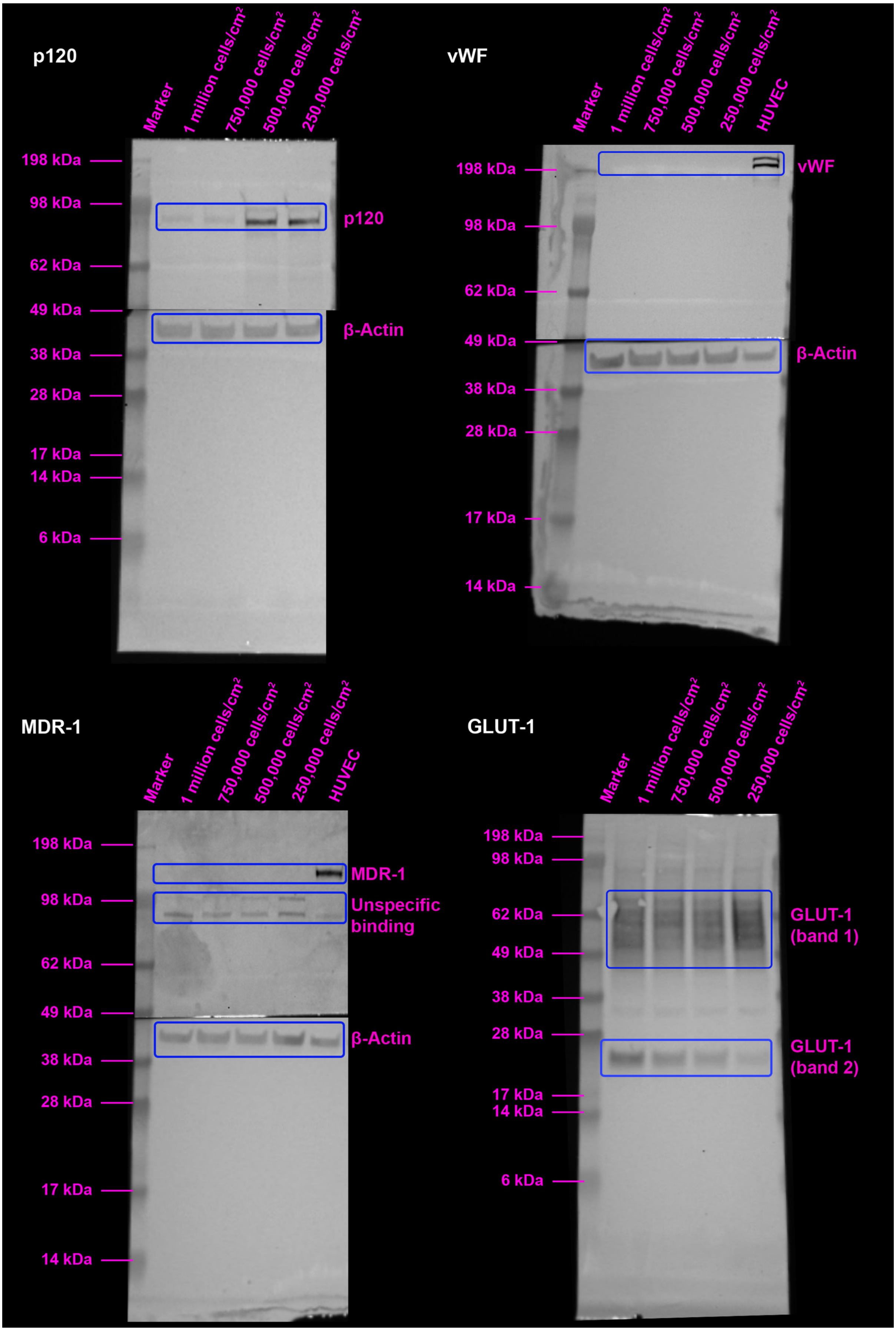

Figure S9. Extended Western blot results of Figure 1 (part 2). Western blot analysis shows the expression of endothelial markers in IMR90-4 hiPSC-derived iBMECs with different plating concentrations (cells were grown on low porosity ThinCert® (#665641) filters for two days).

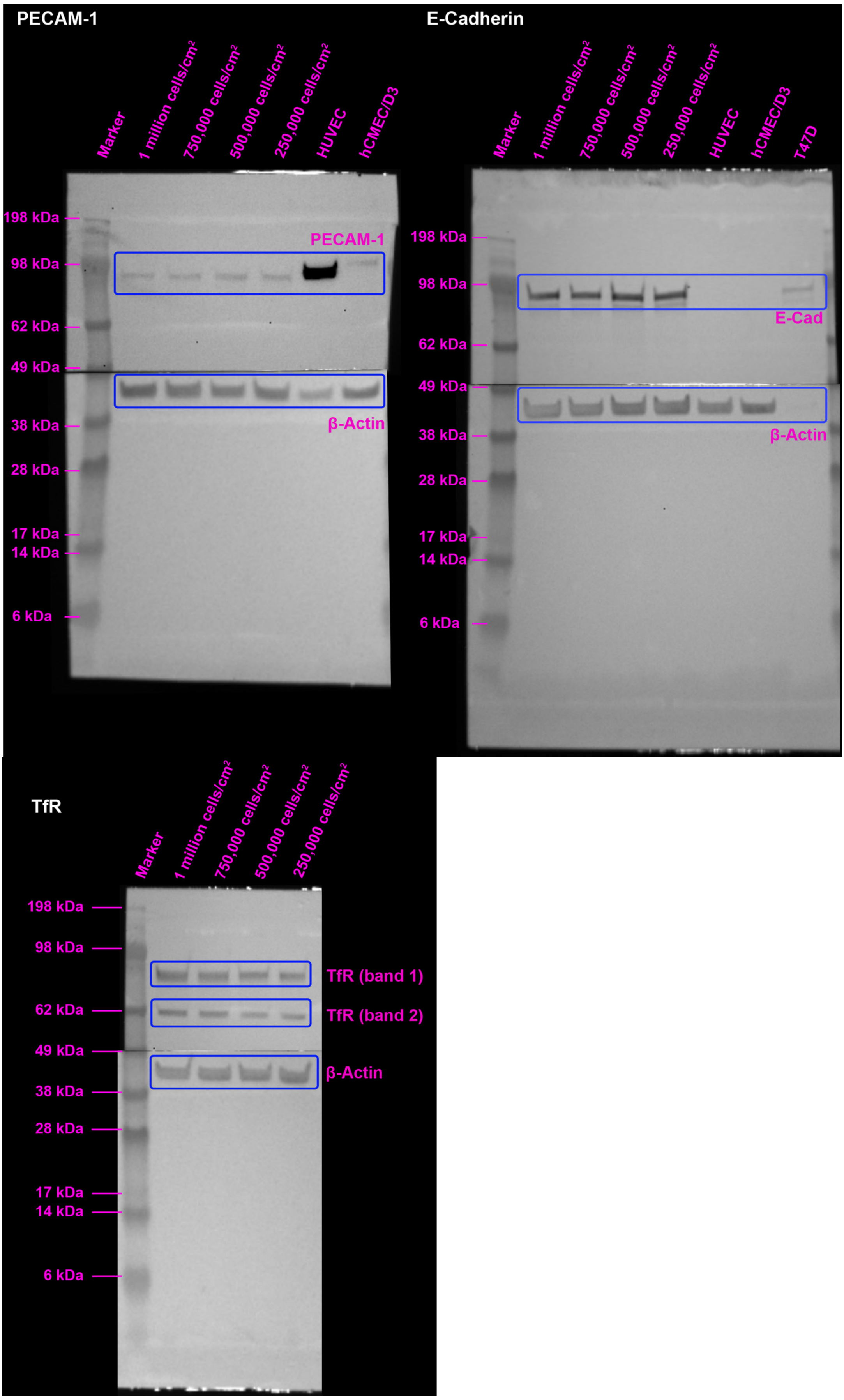

Figure S10. Extended Western blot results of Figure 1 (part 3). Western blot analysis shows the expression of PECAM-1, E-Cadherin, and TfR in IMR90-4 hiPSC-derived iBMECs with different plating concentrations (cells were grown on low porosity ThinCert® (#665641) filters for two days).

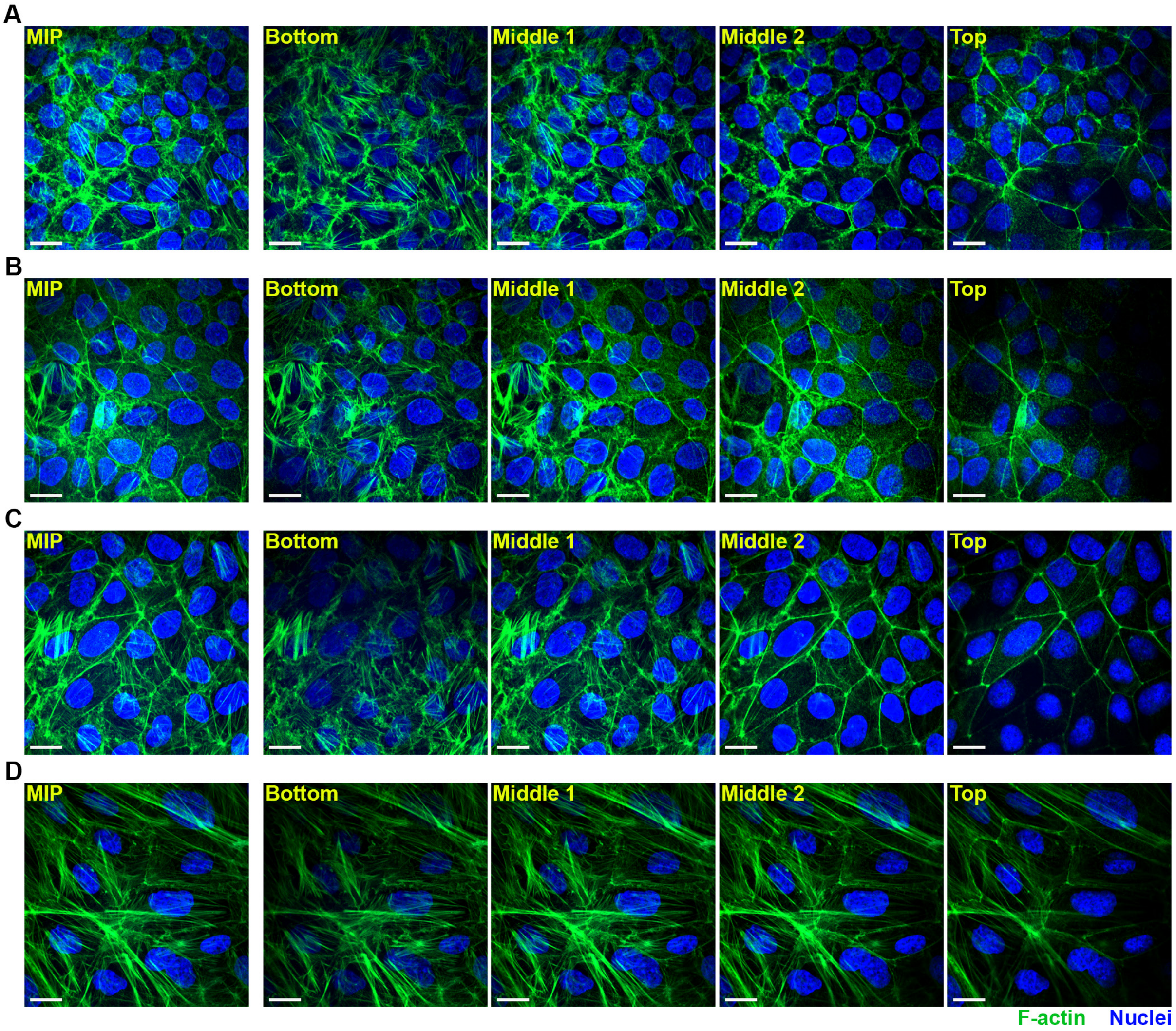

Figure S11. Phalloidin staining of iBMECs derived from IMR90-4 hiPSCs using protocol 1. Representative confocal images showing the expression of F-actin. The iBMECs were grown of low porosity ThinCert® (#665641) filters for 2 days and plated with the following densities (cells/cm^2): 1 million (**A**), 750,000 (**B**), 500,000 (**C**), and 250,000 (**D**). F-actin: green, nuclei: blue. Scale bars represent 20 µm.

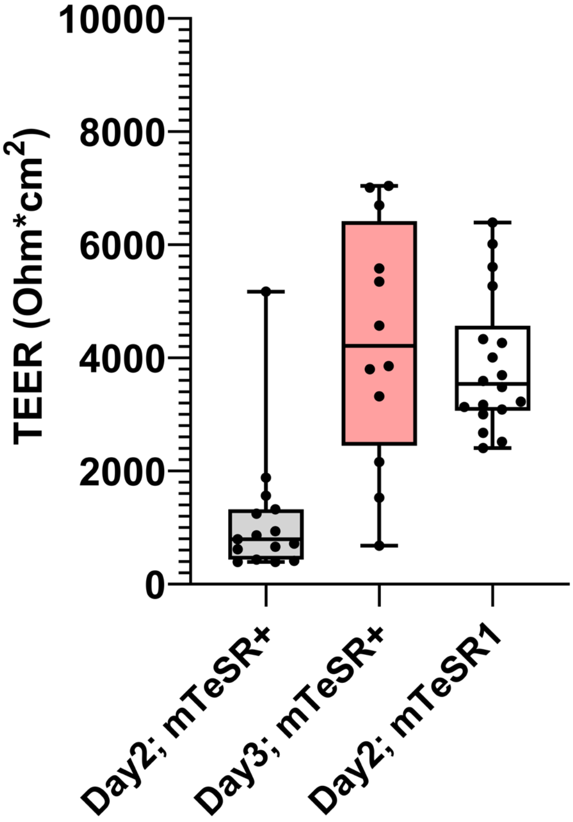

Figure S12. TEER measurement on day 2 or day 3 on filters of iBMECs with 250,000 cells/cm^2 seeding density. The TEER was measured after 20 min equilibration at RT, before media change. Minimum four differentiations with triplicates, box plots with median and min, max. The iBMECs were grown on low porosity ThinCert® *(*#665641) filters for two days.

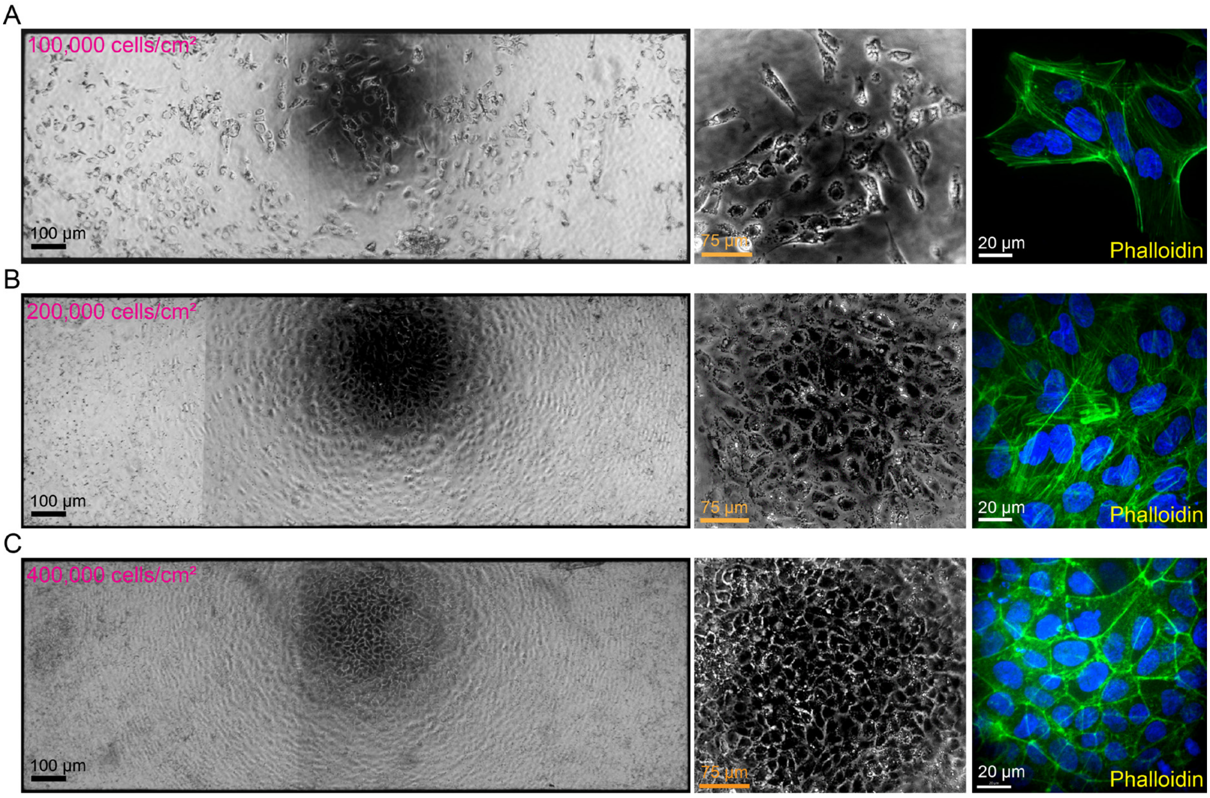

Figure S13. Optimisation of iBMECs seeding density on m-µSiM. The ultrathin NPSN membranes were seeded with differentiated hiPSCs on day 8 and were grown for 2 days. The following seeding densities were used: 100,000 **(A)**, 200,000 **(B)**, and 400,000 **(C)** cells/cm^2. F-actin was labelled with Phalloidin (green), and the nuclei were stained with Hoechst 33342 (blue). Scale bars represent 100 µm, 75 µm, and 20 µm.

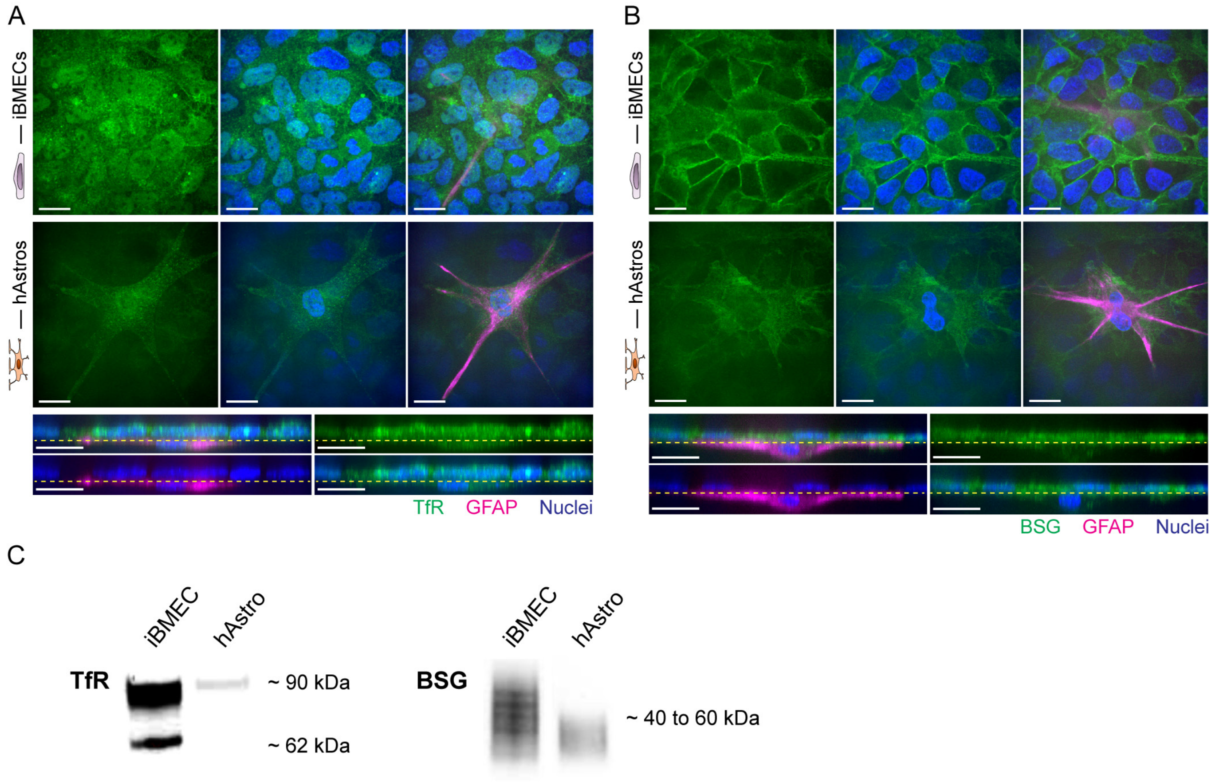

Figure S14. Expression of TfR and BSG receptors in iBMECs and human astrocytes (hAstro). Representative immunofluorescence images **(A, B)** and Western blot **(C)** demonstrate the expression of TfR and BSG in iBMECs and hAstros (much higher expression of both proteins in iBMECs). Immunocytochemistry was done on µSiM-iBMEC co-culture model. Both sides of the membranes were labelled with anti-TfR and anti-BSG Abs to label the receptors in iBMECs and hAstros (green), the hAstros were co-stained with GFAP (magenta), and the nuclei were labelled with Hoechst 33342 (blue). Scale bars represent 20 µm.

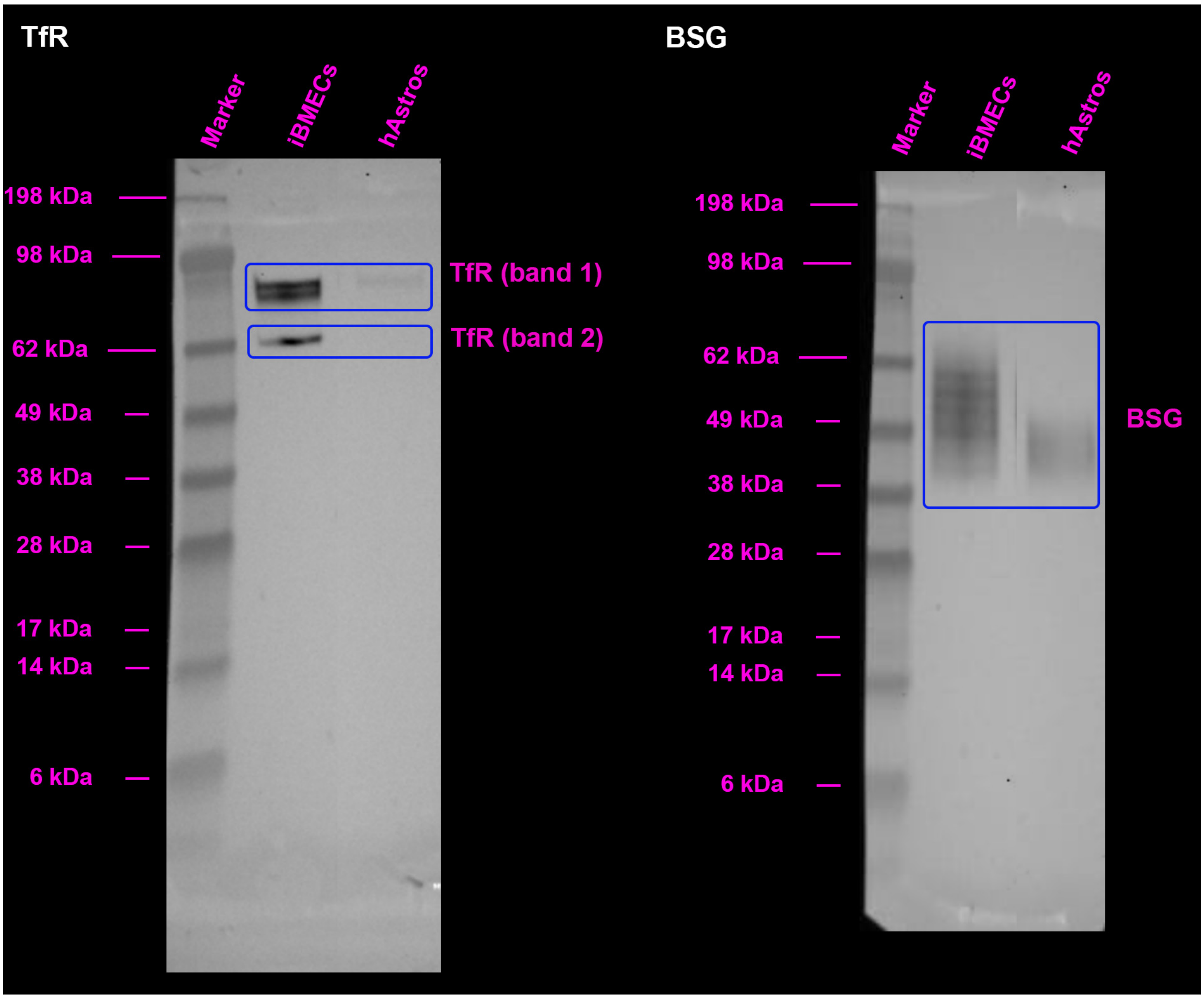

Figure S15. Extended Western blot results of Figure S14. Western blot analysis shows the expression of TfR and BSG in IMR90-4 hiPSC-derived iBMECs and human astrocytes (hAstros).

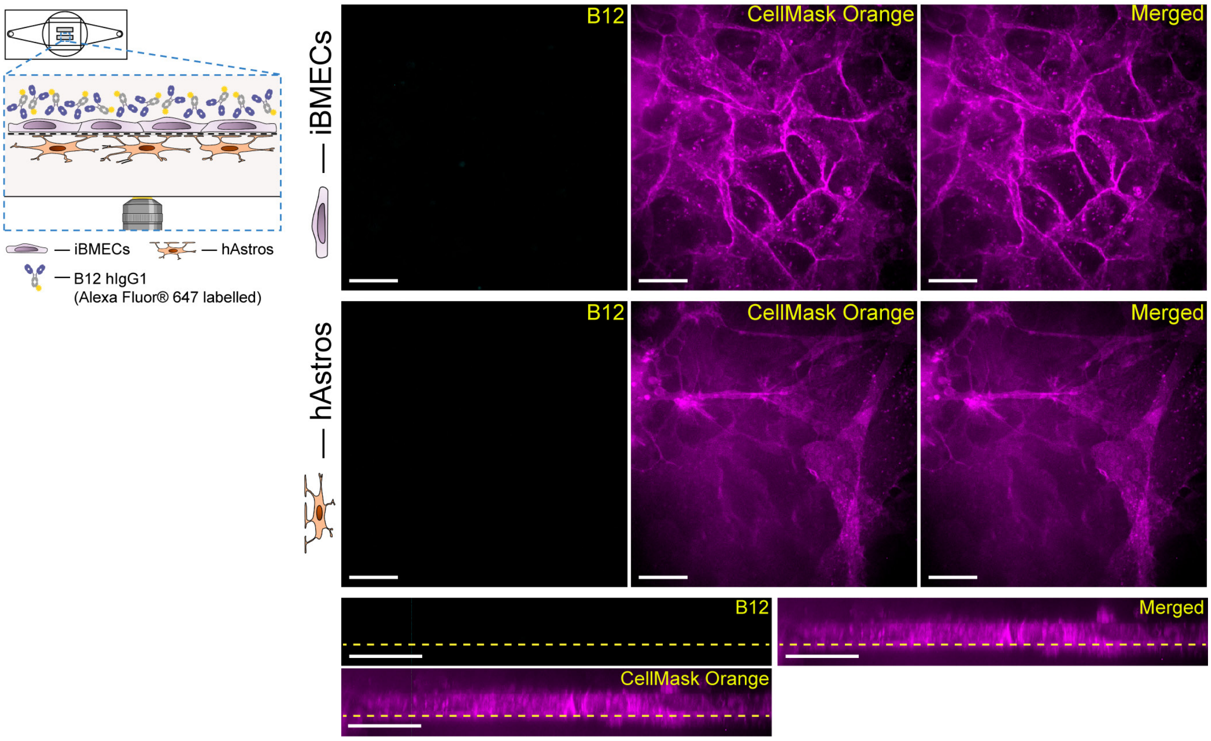

Figure S16. Transcytosis control experiment with B12 hIgG1. Alexa Fluor 647 labelled B12 hIgG1 at a concentration of 500 nM uptake was added to the upper compartment of the m-µSiM for 2 h (pulse) and the cells were imaged 24 h after (chase). Images represent the MIP of a few z-slices from the apical and basolateral compartment and the x-z cross-sectional view of the m-µSiM-iBMEC co-culture. Dashed lines represent the ultrathin NPSN membrane and the scale bars represent 20 µm.

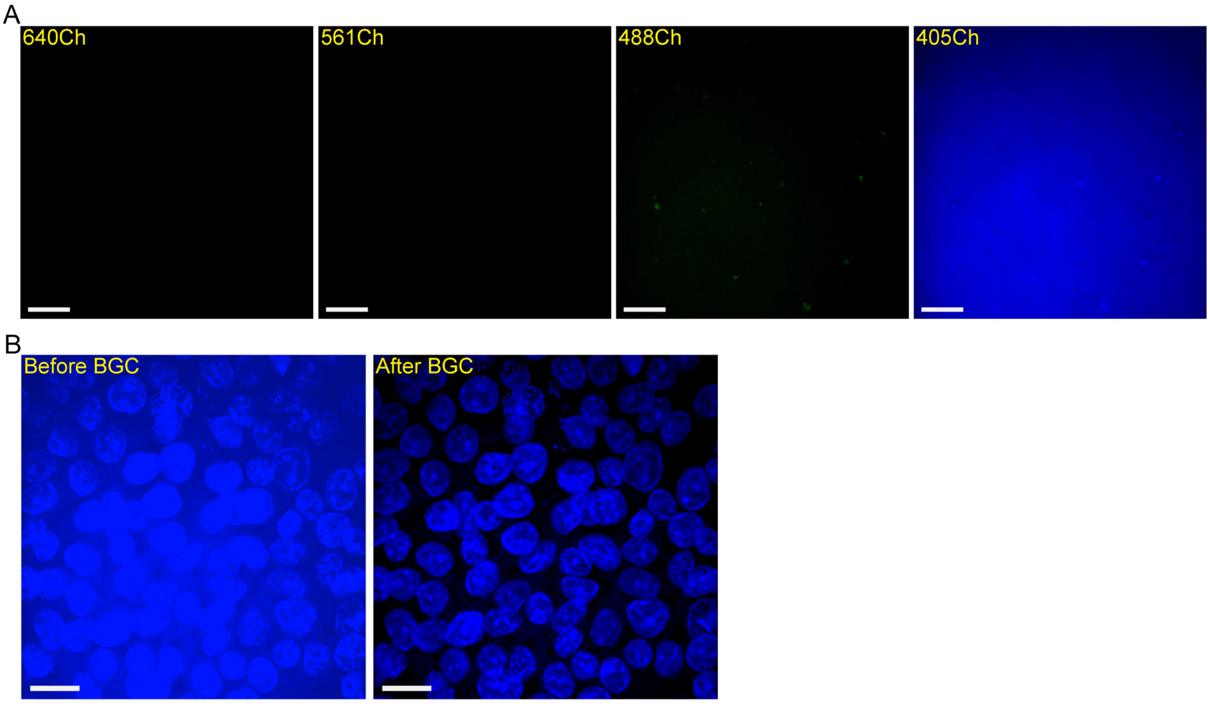

Figure S17. Autofluorescence of Greiner low porosity polyester membrane and the results of top hat background correction (BGC) in the 405 channel (405Ch). Representative maximum intensity projection (MIP) (confocal) images. A) 405Ch: 405 nm excitation laser line, 440/521/607/700 quad-band bandpass emission filter; 488Ch: 488 nm excitation laser line, 525/50 bandpass emission filter; 561Ch: 561 nm excitation laser line, 625/90 bandpass emission filter; 640Ch: 640 nm excitation laser line, 680/42 bandpass emission filter. B) Representative MIP confocal image of 405Ch with Hoechst 33342 stained iBMEC nuclei before and after the top hat background correction using Arivis Vision 4D software. Scale bars represent 20 µm.

References

1. Stebbins, M. J.; Wilson, H. K.; Canfield, S. G.; Qian, T.; Palecek, S. P.; Shusta, E. V. Differentiation and characterization of human pluripotent stem cell-derived brain microvascular endothelial cells. *Methods* **2016,** *101*, 93-102. doi: 10.1016/j.ymeth.2015.10.016

2. Neal, E. H.; Marinelli, N. A.; Shi, Y.; McClatchey, P. M.; Balotin, K. M.; Gullett, D. R.; Hagerla, K. A.; Bowman, A. B.; Ess, K. C.; Wikswo, J. P.; Lippmann, E. S. A Simplified, Fully Defined Differentiation Scheme for Producing Blood-Brain Barrier Endothelial Cells from Human iPSCs. *Stem Cell Reports* **2019,** *12*, 1380-1388. doi: 10.1016/j.stemcr.2019.05.008

3. Stebbins, M. J.; Gastfriend, B. D.; Canfield, S. G.; Lee, M. S.; Richards, D.; Faubion, M. G.; Li, W. J.; Daneman, R.; Palecek, S. P.; Shusta, E. V. Human pluripotent stem cell-derived brain pericyte-like cells induce blood-brain barrier properties. *Sci Adv* **2019,** *5*, eaau7375. doi: 10.1126/sciadv.aau7375

4. Qian, T.; Maguire, S. E.; Canfield, S. G.; Bao, X.; Olson, W. R.; Shusta, E. V.; Palecek, S. P. Directed differentiation of human pluripotent stem cells to blood-brain barrier endothelial cells. *Sci Adv* **2017,** *3*, e1701679. doi: 10.1126/sciadv.1701679
